# Adaptive ER stress promotes mitochondrial remodelling and longevity through PERK-dependent MERCS assembly

**DOI:** 10.1038/s41418-025-01603-7

**Published:** 2025-11-01

**Authors:** Jose C. Casas-Martinez, Qin Xia, Penglin Li, Maria Borja-Gonzalez, Antonio Miranda-Vizuete, Emma McDermott, Peter Dockery, Leo R. Quinlan, Katarzyna Goljanek-Whysall, Afshin Samali, Brian McDonagh

**Affiliations:** 1 Discipline of Physiology, School of Pharmacy and Medical Sciences, University of Galway, Galway, Ireland; 2 Apoptosis Research Centre, University of Galway, Galway, Ireland; 3 Galway RNA Research Cluster, University of Galway, Galway, Ireland; 4 Department of Orthopedics, Tongji Hospital, Tongji Medical College, Huazhong University of Science and Technology, Wuhan, China; 5 Instituto de Biomedicina de Sevilla, IBiS/Hospital Universitario Virgen del Rocío/CSIC/Universidad de Sevilla, Sevilla, Spain; 6 Centre for Microscopy and Imaging, Discipline of Anatomy, University of Galway, Galway, Ireland; 7 Institute of Life Course and Medical Sciences, University of Liverpool, Liverpool, UK; 8 School of Biological and Chemical Sciences, University of Galway, Galway, Ireland

**Keywords:** Ageing, Macroautophagy

## Abstract

The transfer of information and metabolites between the mitochondria and the endoplasmic reticulum (ER) is mediated by mitochondria-ER contact sites (MERCS), allowing adaptations in response to changes in cellular homeostasis. MERCS are dynamic structures essential for maintaining cell homeostasis through the modulation of calcium transfer, redox signalling, lipid transfer, autophagy and mitochondrial dynamics. Under stress conditions such as ER protein misfolding, the Unfolded Protein Response (UPR^ER^) mediates PERK and IRE1 activation, both of which localise at MERCS. Adaptive UPR^ER^ signalling enhances mitochondrial function and calcium import, whereas maladaptive responses lead to excessive calcium influx and apoptosis. In this study, induction of mild acute ER stress with tunicamycin (TM) in myoblasts promoted myogenesis that required PERK for increased MERCS assembly, mitochondrial turnover and function. Similarly, treatment of *C. elegans* embryos with an acute low concentration of TM, promoted an extension in lifespan and health-span. The adaptive ER stress response following a low dose of TM in both myoblasts and *C. elegans*, increased MERCS assembly and activated autophagy machinery, ultimately promoting an increase in mitochondrial remodelling. However, these beneficial adaptations were dependent on the developmental stage, as treatment of myotubes or adult *C. elegans* resulted in a maladaptive response. In both models the adaptations to UPR^ER^ activation were dependent on PERK signalling and its interaction with the UPR^mt^. The results demonstrate PERK is required for the increased mitochondrial ER communication in response to adaptive UPR signalling, promoting mitochondrial remodelling and improved physiological function.

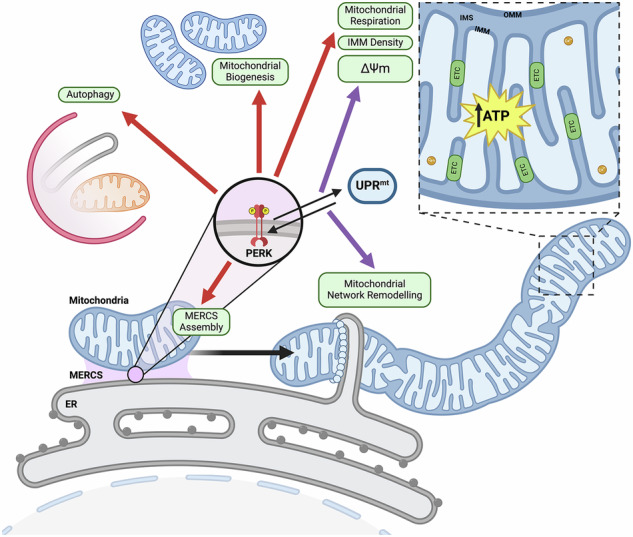

## Introduction

The ER and mitochondria are highly dynamic structures continuously undergoing structural remodelling in response to specific cellular signals. Communication between these organelles can result in physical interactions, called mitochondrial ER contact sites (MERCS), that synergistically affect both organelles [1, 2]. MERCS are dynamic structures that remodel in response to intra- and extra cellular signals, affecting the function of both mitochondria and ER [3–5]. As a result, mitochondrial function is highly sensitive to changes in ER homeostasis and ER stress, transmitted to mitochondria through changes in metabolite communication (Ca^2+^ signalling or lipid exchange) or via stress-responsive signalling pathways [6]. An accumulation of misfolded proteins within the ER can activate the unfolded protein response (UPR^ER^). The activation of the UPR^ER^ can be an adaptive beneficial response, or under conditions of chronic ER stress can be maladaptive, resulting in pro-apoptotic signalling events. Disruption of MERCS assembly and communication between these organelles has been reported in an array of age-related diseases including cancer, neurodegenerative conditions and sarcopenia [7]. Targeting or modulating inter organelle communication by modulating MERCS assembly is a potential therapeutic strategy for various pathophysiological conditions.

The UPR^ER^ is activated following ER stress, mediated by protein kinase RNA-like ER kinase (PERK), inositol-requiring enzyme 1α (IRE1α) and activating transcription factor 6 (ATF6) [8]. Activation of the PERK arm results in phosphorylation of Serine51 of eukaryotic initiation factor 2α (eIF2α) [9], promoting a rapid attenuation of global mRNA translation, reducing the protein load for folding in the ER [10]. Phosphorylated eIF2α increases selective translation of activating transcription factor 4 (ATF4), inducing the translation of ER stress genes related to the restoration of cellular homeostasis [8]. Accumulation of misfolded proteins, protein aggregation and inefficiency in mitochondrial import, results in the activation of the UPR^mt^ by ATF4, activating transcription factor 5 (ATF5) and C/EBP homologous protein (CHOP) [11]. Together they promote the transcription of genes that aid in the recovery of proteostasis, upregulating chaperonins, chaperones, proteases and antioxidant proteins [12].

PERK can modulate mitochondrial function in response to ER stress and is reported to localise at MERCS [2, 13, 14]. PERK activation following ER stress has been demonstrated to be required for acute stress-induced mitochondrial hyperfusion (SIMH) [13]. Mitochondrial morphology can switch from tubular to hyperfused or fragmented, depending on cellular metabolic conditions and in response to stress insults [15]. Mitochondrial dynamics regulated by fusion, fission and biogenesis [16], maintains the efficient diffusion of metabolites and proteins [17] and mtDNA levels [18]. The ER can coordinate mitochondrial dynamics by establishing contact sites between ER tubules and mitochondria [19]. Depending on the severity of the cellular stress, ER-mitochondria signalling can directly affect mitochondrial function leading to either pro-survival or pro-apoptotic signals [20]. An increase in MERCS assembly facilitates Ca^2+^ transfer between the organelles, affecting mitochondrial metabolism due to many Ca^2+^-dependent enzymes within the tricarboxylic acid cycle, while prolonged Ca^2+^ entry can result in the opening of the mPTP, triggering pro-apoptotic signalling [21].

The nematode *Caenorhabditis elegans* is an excellent physiological model for dissecting the mechanisms of adaptive signalling. During ageing, *C. elegans* body wall muscles undergo a decline in mitochondrial dynamics, accumulation of lipids and loss of muscle mass, similar to the ageing process of skeletal muscle from vertebrates [22]. The UPR^ER^ is highly conserved in nematodes, whose function resembles their mammalian orthologues [23]. The activation of UPR^mt^ in *C. elegans* is regulated by the transcription factor ATFS-1 (ATF5 ortholog in *C. elegans)*. ATFS-1 is imported into the mitochondrial matrix by recognition of its mitochondrial targeting sequence by TOM/TIM, where it is degraded by the matrix-localized protease LON [11]. Perturbations of mitochondrial homeostasis impair ATFS-1 mitochondrial import and promotes the nuclear translocation of ATFS-1, resulting in the activation of the UPR^mt^ [24].

This study investigated the role of adaptive UPR^ER^ signalling at an early developmental stage in a cell model of myogenesis and using the whole organism *C. elegans*. Mechanistically adaptive UPR^ER^ signalling resulted in increased MERCS formation, increased mitochondrial dynamics and function that improved myogenesis and healthspan of *C. elegans*. PERK/PEK-1 was identified as the signalling mechanism underlying the adaptive UPR^ER^ response and resulted in downstream activation of ATF5/ATFS-1. The results provide novel insights into the potential of targeting UPR^ER^ signalling and MERCS assembly to develop therapeutic strategies to mitigate age-related conditions and neurodegenerative diseases.

## Materials and methods

### *C. elegans* strains and culture conditions

*C. elegans* strains used in this study are listed in Supplementary Table 4. Nematode Growth Medium (NGM) was used to maintain worms at 20° C.

### RNA interference

RNAi downregulation was performed by feeding worms with HT115 bacteria expressing the dsRNA of the desired target gene. A single colony of the corresponding bacteria was inoculated into LB, supplemented with ampicillin (100 μg/ml), and incubated at 37° C overnight. NGM plates containing ampicillin (100 μg/ml) and IPTG (1 mM) were seeded with 100 μl of the bacterial culture and incubated at 37° C for 48 h. A synchronised population of worms were grown on plates containing the corresponding RNAi.

### Cell lines culture conditions

C2C12 cells were grown in standard growth medium (GM) containing DMEM with 10% FBS and 1% penicillin/streptomycin_._ Cells were differentiated using DMEM containing 2% horse serum (DM) and allowed differentiate for up to 7 days. Myotube area, diameter and fusion index were quantified as previously described [25]. For siRNA, cells were treated with 50 nM siRNA for 5 h in serum-free DMEM. Media was refreshed with GM, and cells were allowed to recover overnight. Knockdown efficiency was validated by Western blotting of the proteins.

### Cell and *C. elegans* treatments

TM treatment of C2C12 skeletal muscle myoblasts was performed when cells reached 70–80% confluency. Cells were treated with a range of TM concentrations for 8 h, subsequently cells were allowed to recover in DM overnight, myoblasts were prepared for analysis or differentiated in DM for 7 days. TG treatment of myoblast was performed following the same procedure as with the TM treatment. For *C. elegans* a synchronised population of eggs were obtained by bleaching. S medium was prepared using a pellet of OP50 resuspended in M9 buffer and harvested eggs were treated with TM diluted in S medium for 24 h [26]. The same procedure was followed for the treatment with thapsigargin (TG) and N-acetyl cysteine (NAC). After treatment, larvae were transferred to NGM plates and allowed to grow until adulthood.

### Western blotting

C2C12 myoblasts were homogenised on ice in lysis buffer (150 mM NaCl, 20 mM Tris pH 7.5, 1 mM EDTA pH 8.3, 0.5% SDS, 1% Triton) supplemented with protease and phosphatase inhibitors. Samples were prepared by scraping of cells, followed by 30 s homogenisation. Protein extracts were quantified using the Bradford assay. Protein samples were diluted with Laemmli buffer, 20 μg of protein was loaded onto 12% SDS PAGE gels for Western blots. The proteins were transferred using a semi-dry blotter and the membrane was stained with Ponceau S to ensure complete transfer and for normalisation. Membranes were blocked in 5% milk in TBS-T or 3% BSA in TBS-T (for phosphorylated proteins). Subsequently, membranes were incubated with primary antibodies (as listed) at a 1:1000 dilution overnight, following washing membranes were incubated with secondary antibodies at a 1:10000 dilution in TBS-T in the dark for 1 h. Images were captured using the Odyssey FC imaging system. Quantification of selected bands was performed using densitometry macros in ImageJ and normalised using total protein staining. Uncropped Western blots are displayed in the Supplementary data file.

### PCR analysis

TRIZol was added directly onto the cells and transferred into 1.5 ml Eppendorf tubes. For *C. elegans* samples, age-synchronised worms were incubated with TRIZol (500 µl/NGM plate) and vortexed for 30 s. Chloroform was added, tube was shaken vigorously by hand for 15 s and incubated for 5 min. RNA precipitation was achieved by adding 100% isopropanol to the aqueous phase and incubated at –20° C overnight. RNA pellets were washed by adding 80% ethanol, supernatant was discarded and the tubes allowed to air dry. RNA was reconstituted in 10 μl of RNase-free H_2_O. Samples were analysed using the NanoDrop2000 spectrophotometer. cDNA was synthesised using 500 ng of RNA. For cDNA synthesis all samples used 500 ng of RNA, 1 ml random hexamers, 4 ml RT Buffer, 2 ml DTT, 1 ml dNTP, 1 ml Superscript II and 1 ml Ribolock. The samples were run in a thermocycler for 10 min at 65 °C and 60 min at 42 °C, samples were diluted 10× (180 μL H_2_O) and kept at –20 °C until needed. For the PCR analysis of XBP1 splicing, a PCR master mix was prepared containing: 1 μL of a primer mix Fwd+Rev (each 10 μM), 4 μL of H_2_O, and 6.25 μL of RedTaq for each sample. 12.25 μL of master mix was added to each of the reaction PCR tubes and then 1.25 μL of cDNA into each tube (total volume of 15 μL). Tubes were placed in a thermocycler with the following program: [*Xbp-1u* and *Xbp-1s*] 94 °C for 3 min, 94 °C for 30 s, 58 °C for 30 s, 72 °C for 30 s, 72 °C for 7 min (repeat steps 2 to 4 for 35 cycles), followed by 4 °C until analysis. RT-qPCR was used to study gene expression was performed using SybrGreen qPCR as previously described [25]. Expression relative to the corresponding housekeeper gene was calculated using the ΔΔCt method.

### Immunohistochemistry

Mitochondrial staining of myoblasts was performed following overnight recovery in DM following TM treatment. Medium was aspirated and cells were rinsed with PBS. Myoblasts were then incubated with mitochondrial dyes for content (200 nM MitoTracker green), and membrane potential (100 nM TMRM) or ROS (5 μM MitoSox) diluted in serum-free DMEM for 20 min. After a final PBS wash, images were captured. Regions within each cell showing staining fluorescence signals were identified as mitochondrial. CTCF was calculated by the integrated density—(area of cell X Mean fluorescence of background readings) [27]. MF20 immunostaining was performed after 7 days of differentiation. Following fixation, samples were blocked and incubated overnight with 1:500 primary MF20 antibody in 2% horse serum in PBS and 1:2000 of secondary antibody solution (anti-mouse 488) in 2% horse serum in PBS. Cells were incubated for 10 min in the dark at RT with 1:10,000 DAPI in PBS. Myotube diameter was determined by measuring the thickest diameter of each myotube. The myotube area fraction measures the percentage of the total image area that was occupied by myotubes. The fusion index, measures the fraction of the nuclei that are inside the myotubes and the total nuclei number, indicating their regenerative potential.

### Viability assay

The live/dead assay used a combination of ethidium bromide and acridine orange to distinguish between viable and nonviable cells. Ethidium bromide and acridine orange at 1:1000 in PBS, was added to the cells and incubated at room temperature for 5 min. After incubation, the cells were imaged using an EVOS 7000 microscope, and the images were analysed with ImageJ software to quantify the number of live, dead, and necrotic cells. Each experimental group consisted of 3 replicates, with 4 images captured per replicate. Acridine orange stained all cells green, red-stained nuclei indicated cell death.

### Mitochondrial respiration rates

C2C12 myoblasts were plated in triplicate in XF HS Mini Analyzer plates at a density of 8000 cells per well in a total volume of 200 μl per well and incubated for 24 h. Cells were washed once with XF Real-Time Mito-Stress Assay Medium at 37 °C. The concentrations of chemicals were optimised and based on the supplier instructions, 2 μM Oligomycin, 2 μM FCCP, and 1 μM antimycin A. For measuring mitochondrial respiration rates of *C. elegans*, the compounds (10 μM FCCP and 40 mM sodium azide) were added to the hydrated cartridge. Three replicates for each condition were each well containing 8–13 worms and transferred to XF HS Mini Analyzer plate before starting the calibration process, *C. elegans* procedures were performed at 20 °C as previously described [28].

### Mass spectrometry

Proteomics was performed to indicate the global label-free relative abundance of proteins. Protein extracts were prepared using a hand homogeniser. Samples were incubated with trypsin overnight at 37 °C, digestion was stopped and RapiGest removed by acidification [29]. Mass spectrometry analysis was performed at the proteomics facility NICB at Dublin City University. An UltiMate 3000 RSLCnano LC system was coupled to an Orbitrap Fusion^TM^ mass spectrometer, LC-MS/MS analysis was performed as described in [30].

Mass spectrometry data was analysed for global changes in protein abundance. Raw files were processed using MaxQuant (v1.6.0.16) against a *Mus musculus* protein database (UniProtKB/Swiss-Prot/TrEMBL, n sequences). Results were filtered at 0.01 false discovery rate (FDR) at both peptide and protein levels. Quantification was performed using Prostar software [31]. Proteins with fewer than three valid values in at least one experimental condition were filtered out. Proteins with a p-value < 0.01 and Log_2_FC ratio >0.58 or <–0.58 (FC > 1.5) giving an estimated 6.5% FDR by Benjamini-Hochberg correction. Volcano plots were generated using R 4.3.2 and the package ggplot2 3.4.4. Enrichment analysis and dot-plots were obtained with ShinyGO.

### RNA sequencing and bioinformatic analysis

Total RNA was extracted from 4 individual NGM plates containing a population of *C. elegans*. A NanoDrop 2000 was used to determine each sample’s purity and concentration. Samples were sent to Novogene for bulk RNA-sequencing on Illumina platforms. Data analysis was performed by Novogene including sample and data quality control. Alignment was performed using the reference *C. elegans* genome with HISAT2 2.0.5, gene expression quantification with featureCounts v1.5.0-p3, differential expression analysis with DESeq2 v1.20.0 and gene set enrichment analysis was performed with GSEA v4.3.2. The differential expression was obtained by normalisation of the raw readcount, the hypothesis test’s probability (p-value) was calculated by a statistical model and finally multiple hypothesis test corrections were used to obtain FDR values. The Multiple Testing online tool was used to calculate the FDR of the comparison [32]. The selected differential expression analyses were *EMBRYO CNT vs EMBRYO TM* and *L4 CNT vs L4 TM*. The screening criteria for differential genes used were Log_2_FC > 1 and p adj < 0.05. R 4.3.2 was used for generation of volcano plots with ggplot2 3.4.4. Enrichment analysis and dot-plots were obtained with ShinyGO. PathVisio 3.3.0 was used to highlight altered pathways from detected transcripts.

### Ca^2+^ imaging

Cells were washed with Ca^2+^-containing medium (NaCl 135 mM, KCl 5 mM, MgCl_2_ 1 mM, CaCl_2_ 2 mM, HEPES, 10 mM and Glucose 1 mM, pH 7.45) and loaded with 2 μM Fluo-4/AM (5 μM) for 40 min. Cells were washed and left in the dark for an additional 15 min to allow for the de-esterification of Fluo-4/AM. Media was replaced and images were recorded over a 20 min video at 0.5 Hz. To assess Store-Operated-Ca^2+^-Entry (SOCE), an indicator of Ca^2+^ levels in the ER, the classical thapsigargin/Ca^2+^ re-addition protocol was followed [33]. Briefly, cells were left for 5 min in Ca^2+^-containing medium, media was replaced with Ca^2+^-free medium. After 2 min, 1 μM thapsigargin was added and after 8 min media was replaced with Ca^2^-containing medium. Timelapse videos were processed and analysed using FluoroSNAP in MATLAB as described in [34, 35].

### Transmission electron microscopy

C2C12 myoblasts, myotubes and *C. elegans* were fixed using 2% glutaraldehyde and 2% paraformaldehyde in 0.1 M sodium cacodylate buffer at pH 7.2. Following fixation, the buffer was replaced with 1% osmium tetroxide in 0.1 M sodium cacodylate buffer (pH 7.2). Subsequently, samples underwent dehydration using a series of ethanol concentrations and were infiltrated with resin-acetone mixtures overnight, starting with a 50:50 mixture, a 75:25 mixture, and finally 100% resin. Ultra-thin sections (70–90 nm) were cut in a sagittal plane using a diamond knife and mounted onto 3 mm copper grids. The grids were then counterstained for 5 min with 2% uranyl acetate and 5 min with 2% lead citrate, after that they were examined under a Hitachi 7500 TEM microscope at a magnification of ×10,000, ×25,000 and ×60,000. Traditional point-counting methods assessed cell and organelle composition and parameters were calculated following formulas described in [36].

### Lifespan assays

One hundred L4 worms were transferred to NGM plates supplemented with 10 mM 5-fluorouracil (5-FU). At L4 stage, worms were transferred to the 5-FU supplemented NGM plates. Lifespan assay was initiated on day 1 of adulthood and their viability was scored every 2 days. Worms were considered dead when the animal did not respond to gentle touch with a pick, animals were censored when they died by desiccation, ruptured vulva or internal hatching.

### Oxidative stress survival assays

On day 1 of adulthood, 50 worms were transferred to a 24-well plate, each well containing 250 μl of M9 buffer and a final concentration of 10 mM NaAsO_2_ or 100 mM of Paraquat in M9 buffer. Viability was scored every hour and the worms were considered dead when the animal did not respond to gentle touch with a pick [37]. For the tBuOOH stress assay NGM plates containing 15.4 mM tBuOOH were used. 50 worms were transferred to the test plates along with some OP50 to avoid their escape from the plate (during the first hour of the assay the worms were monitored every 10 min to avoid desiccation when trying to escape the plate). Viability was scored every hour and the worms were considered dead when the animal did not respond to gentle touch with a pick [38].

### *C. elegans* imaging

For imaging mitochondrial parameters, worms were incubated with either of 2.5 μM MitoTracker Red CMXRos or 10 μM MitoSOX Red, diluted in M9 buffer, for 10 min or 1 h, respectively. Worms were transferred to seeded NGM plates and allowed to forage for 2 h to prevent stain accumulation. Subsequently, worms were immobilised on unseeded NGM plates using a drop of 20 mM Levamisole. All steps were conducted in darkness. ImageJ was used to assess the GFP CTCF for each worm. In all strains including length measurements, 15 worms per replicate, for a total of three replicates were immobilised on unseeded NGM plates and imaged. The length measurements were assessed using the *segmented line* tool from ImageJ.

LysoSensor green and LysoTracker staining were conducted on *C. elegans* by soaking in M9 buffer containing 10 mM LysoSensor Green DND 189 and LysoTracker red DND 99 for 1 h. Worms were transferred to fresh NGM plates with OP50 and allowed to recover for 1 h. Imaging was conducted at X60 magnification and the LSG/LTR fluorescence in the intestine area for each worm was analysed using ImageJ. Oil red O staining was conducted as previously reported [39]. Animals were mounted on glass slides. Oil red O was quantified from colour images using the level of excess red intensity in the red channel compared to the blue and green channels as described [40].

The physiological stress reporters *hsp-4p::GFP, hsp-6p::GFP and gst-4p::GFP* were imaged using the standard procedure adapted from [38]. Prior to the imaging induction of ER or mitochondrial stress was performed. L4 *hsp-4p::GFP* animals were transferred to plates with 25 ng/μl TM supplemented OP50 plates for 16 h. L4 *hsp-6p::GFP* animals were treated with 50 μM CCCP supplemented OP50 plates for 8 h. Day 1 *gst-4p::GFP* animals were treated with 50 µM Paraquat in M9 Buffer for 2 h, followed by a recovery on NGM plates for 2 h. The *myo-3::mitoGFP* reporter strain for muscle mitochondrial morphology was imaged using ×60 magnification. On day 1 of adulthood, 45 worms per condition were immobilised with 20 mM Levamisole on a 4% agar pad slide. Mitochondrial morphology between the pharynx and vulva was evaluated based on three classifications: filamentous (showing filamentous mitochondrial networks), punctate (displaying mostly fragmented mitochondria), and intermediate (exhibiting isolated mitochondrial networks) [41]. Mitophagy was assessed using the *p*_*myo-3*_*TOMM-20::Rosella* (a gift from the Tavernarakis lab) strain. Imaging was conducted at X40 magnification and the CTCF of green and red fluorescence in the head region of each worm, selecting a muscle-specific area just anterior to the intestine to avoid gut autofluorescence, was analysed using ImageJ.

### CeleST swimming activity assay

Swimming behaviour of the different *C. elegans* strains was assessed at day 1 of adulthood or as indicated. On the day of the assay, a glass slide with a 10 mm pre-printed ring was prepared and loaded with 50 μl M9 Buffer. Five worms were picked and placed into the swimming area, for 45 animals per condition. Worms were allowed to settle in the M9 Buffer for 20 s before starting recording. 30 s movies with ~16 frames per second of the animals were taken using a Nikon LV-TV microscope with a OPTIKA C–P20CM camera and processed by CeleST software [42].

### Statistical analysis

Statistical data were represented as mean ± SEM. For comparisons of means between two groups a two-tailed unpaired Student’s *t* test was performed (data normality validated using GraphPad prism). For comparisons involving more than two groups, one-way or two-way analysis of variance (ANOVA) was used. Lifespan and survival assay data were analysed using Kaplan–Meier survival analysis with a Log-rank test when comparing two conditions. For multiple comparison survival analysis, χ^2^ and Bonferroni corrected p-value were calculated for each condition, using the online platform for statistical analysis of survival data, OASIS 2 [43]. Chi-square test was used to compare the distribution into multiple categories. Prism version 9.5 software package for MacOS was used for the statistical analysis. A p-value < 0.05 was considered statistically significant and the significance indicators were assigned: *p ≤ 0.05,**p ≤ 0.01,***p ≤ 0.001 and ****p ≤ 0.0001.

## Results

### PERK promotes myogenesis in myoblasts subjected to physiological levels of ER stressors

**Fig. 1:**
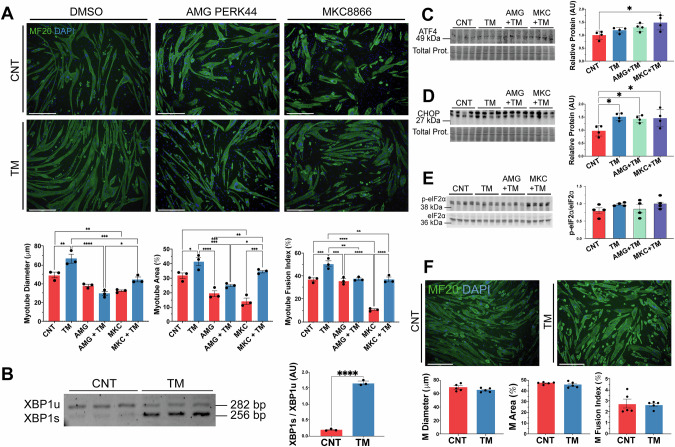
Treatment of C2C12 myoblasts with an acute low concentration of the ER stressor tunicamycin (TM) promotes myogenesis. **A** MF20 immunostaining following TM 0.2 μg/mL treatment of myoblasts resulted in an increase in myotube diameter, area and fusion index. Mean ± SEM; *p ≤ 0.05, **p ≤ 0.01, ***p ≤ 0.001 and ****p ≤ 0.0001 One-way ANOVA. **B** PCR analysis of mRNA expression levels of XBP1s/XBP1u in TM-treated myoblasts. Each lane represents independent biological replicates, data represented as mean ± SEM; ****p ≤ 0.0001 Student’s *t* test. **C–E** Western blot of ATF4, CHOP and p-EIF2*α*/EIF2*α* in TM-treated myoblasts. Each lane represents independent biological replicates, data represented as mean ± SEM; *p ≤ 0.05 and **p ≤ 0.01 One-way ANOVA. **F** MF20 immunostaining following TM treatment 0.2 μg/mL of myotubes, n = 5. Scale 275 μm. Mean ± SEM; Student’s *t* test.

To investigate the role of adaptive UPR^ER^ during myogenesis, C2C12 myoblasts were exposed to a range of concentrations of the ER stressor tunicamycin (TM) for 8 h, followed by differentiation into myotubes (Supplementary Fig. 1A). Notably, treatment of myoblasts with 0.2 μg/ml TM significantly enhanced the myogenic potential of myoblasts. However, concentrations above 0.4 μg/ml were detrimental and promoted cell death (Supplementary Fig. 2A). To determine if the response was an ER stress response or specific to TM, the SERCA inhibitor thapsigargin (TG) was also used. A low concentration of TG (10 nM) also promoted an increase in myogenic parameters (Supplementary Fig. 2B). Inhibition of PERK with AMG PERK44 (AMG) resulted in no difference in myogenesis parameters compared to controls (Fig. 1A). Furthermore, the combined AMG + TM treatment group (AMG + TM) exhibited no change when compared to the AMG group (Fig. 1A). However, inhibition of IRE1α with MKC8866 (MKC) led to a significant decrease in all the parameters compared to controls (Fig. 1A). Nevertheless, the MKC + TM group exhibited an increase in myogenic parameters compared to MKC alone (Fig. 1A). Interestingly, TM-treated myoblasts exhibited a significant decrease in cell viability, with an even greater decrease observed in the AMG + TM group (Supplementary Fig. 2C).

PCR analysis confirmed activation of the IRE1α arm of the UPR^ER^, with a shift toward the spliced, active form of XBP1 (XBP1s) following TM treatment (Fig. 1B). Western blot analysis of UPR^ER^ markers revealed no significant changes in the ER chaperone GRP78, though there was an increasing trend in eIF2α phosphorylation (Supplementary Fig. 2D). There was an increase in the levels of ATF4 in MKC + TM-treated cells, and increasing trend in eIF2α phosphorylation, while CHOP increased following TM treatment and was elevated in both AMG + TM and MKC + TM groups (Fig. 1C–E). To evaluate the different UPR pathways, we performed a qPCR analysis of markers of the mammalian UPR^ER^ (*Hspa5*, *Dnajc3*, *Ddit3*, *Herpud1*, *Pdia4* and *Edem1*) [44] and UPR^mt^ (*Atf5, Lonp1, Hspe1, Hspa1a, Hspd1* and *Hsp90aa1*) [45]. A similar response in all UPR^ER^ markers was observed, a slight increase in gene expression with the TM treatment, however there was a significant increase in levels of UPR^ER^ genes following TM treatment in combination with the PERK inhibitor (Supplementary Fig. 2E). The expression of UPR^mt^ genes *Atf5* and *Lonp1*, follow the same pattern as the UPR^ER^ markers, however, the gene expression of *Hspe1, Hspa1a, Hspd1* and *Hsp90aa1* did not change (Supplementary Fig. 2F). Similar to inhibition of PERK with AMG44, knock-down of PERK (siPERK) in myoblasts decreased myotube diameter following differentiation. Knock-down of PERK or CHOP combined with the TM treatment, exhibited a reduction in myotube area and fusion index compared to siRNA CNT (Supplementary Fig. 2E). Additionally, TM treatment of differentiated, untreated myotubes did not result in any significant differences in the myotube parameters (Fig. 1F). Collectively, these findings demonstrate that inducing mild ER stress in myoblasts improved myogenesis and identified an essential role of PERK in adaptive UPR signalling.

### PERK promotes MERCS assembly and mitochondrial adaptations during adaptive UPR

**Fig. 2:**
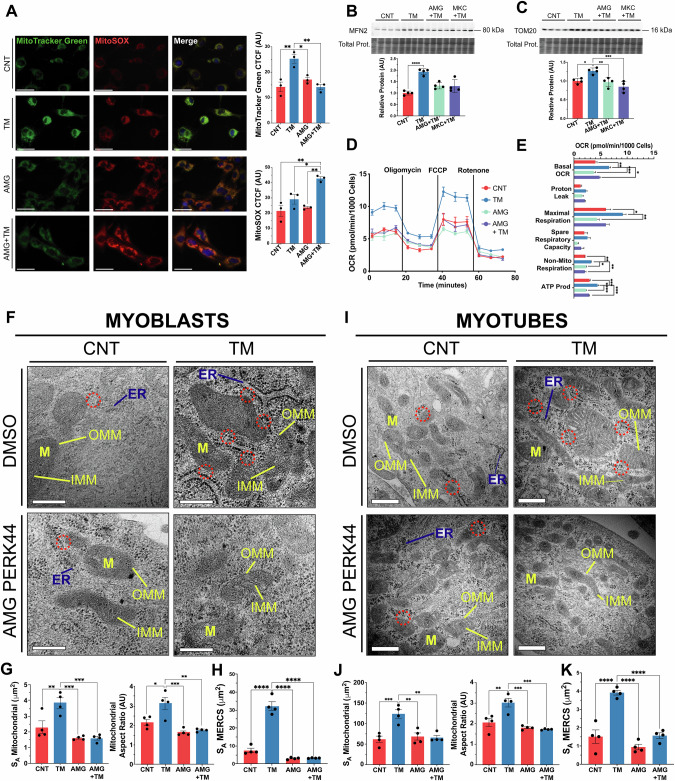
Adaptive UPR^ER^ signalling increases mitochondrial capacity and MERCS assembly that depends on the PERK arm of the UPR^ER^. **A** TM treatment increased mitochondrial content (MitoTracker Green) but did not affect MitoSOX staining; n = 3. Scale 40 μm. Mean ± SEM; * p ≤ 0.05 and **p ≤ 0.01 One-way ANOVA. **B**, **C** Increased levels of MFN2 and TOM20 in TM-treated myoblasts. Each lane represents independent biological replicates, data represented as mean ± SEM; *p ≤ 0.05 and **p ≤ 0.01 One-way ANOVA. **D**, **E** TM treatment of myoblasts increased basal and maximal respiration rate, ATP production and non-mitochondrial respiration. Mean ± SEM; *p ≤ 0.05, **p ≤ 0.01, ***p ≤ 0.001 and ****p ≤ 0.0001 One-way ANOVA. **F**, **G**, **H** Representative TEM images of sections of myoblasts. Mitochondrial surface area, aspect ratio and MERCS surface area increased with the TM treatment. PERK inhibition resulted in no differences compared to the control.; n = 4. Scale 200 nm. Mean ± SEM; *p ≤ 0.05, **p ≤ 0.01, ***p ≤ 0.001 and ****p ≤ 0.0001 One-way ANOVA. **I**, **J**, **K** Representative TEM images of sections from myotubes following TM treatment of myoblasts demonstrating the increase in mitochondrial surface area, aspect ratio and MERCS surface area was maintained in myotubes following TM treatment.; n = 4. Scale 200 nm. Mean ± SEM; *p ≤ 0.05, **p ≤ 0.01, ***p ≤ 0.001 and ****p ≤ 0.0001 One-way ANOVA.

The effects of TM treatment (0.2 μg/ml for 8 h) and/or PERK inhibition on mitochondrial content and function were evaluated in myoblasts (Fig. 1A). MitoTracker Green staining was used to assess mitochondrial content and increased with TM treatment. No differences were observed between the AMG group and the controls (Fig. 2A). Mitochondrial ROS was assessed using MitoSOX staining and there was no change in MitoSOX staining in the TM and AMG groups. However, an increase in fluorescence was observed in the AMG + TM group (Fig. 2A). Additionally, MitoTracker Green and TMRE staining was used to assess mitochondrial membrane potential (ΔΨm), there was a significant increase in both MitoTracker Green and TMRE staining following TM treatment. The ratio of the intensities indicated that TM treatment promoted mitochondrial biogenesis and enhanced ΔΨm (Supplementary Fig. 2F). Both the mitochondrial fusion protein MFN2, which plays a role in mitochondrial-ER tethering [46, 47], and the mitochondrial content indicator TOM20 increased with TM treatment (Fig. 2B, C). The oxygen consumption rate of C2C12 myoblasts demonstrated enhanced basal and maximal respiration, non-mitochondrial respiration, and ATP production in TM-treated cells compared to controls, AMG, and AMG + TM groups (Fig. 2D, E). Furthermore, transmission electron microscopy (TEM) organelle ultrastructure analysis of myoblasts demonstrated an increase in the membrane surface area of mitochondria (S_A_), mitochondrial elongation (aspect ratio) following TM treatment; these adaptations were blocked by PERK inhibition (Fig. 2F, G). Notably, these mitochondrial adaptations were preserved following differentiation into myotubes (Fig. 2I, J). Additional parameters, including mitochondrial volume fraction, cristae membrane density (S_A_ IMM/OMM) and ER surface area, increased in both myoblasts and subsequent myotubes following TM treatment (Supplementary Fig. 2G). The evaluation of MERCS surface area demonstrated an expansion of mitochondrial and ER membranes that are in close proximity following TM treatment. The formation of these expanded subdomains was blocked by inhibition of PERK (Fig. 2F–H). The increase in MERCS surface area was sustained after differentiation into myotubes (Fig. 2I–K). Together, the data demonstrates that PERK is required during adaptive UPR^ER^ to form MERCS and increase mitochondrial content and function in myoblasts, which were maintained following differentiation into myotubes.

### PERK is required for adaptive UPR^ER^ activation of autophagy and mitophagy

**Fig. 3:**
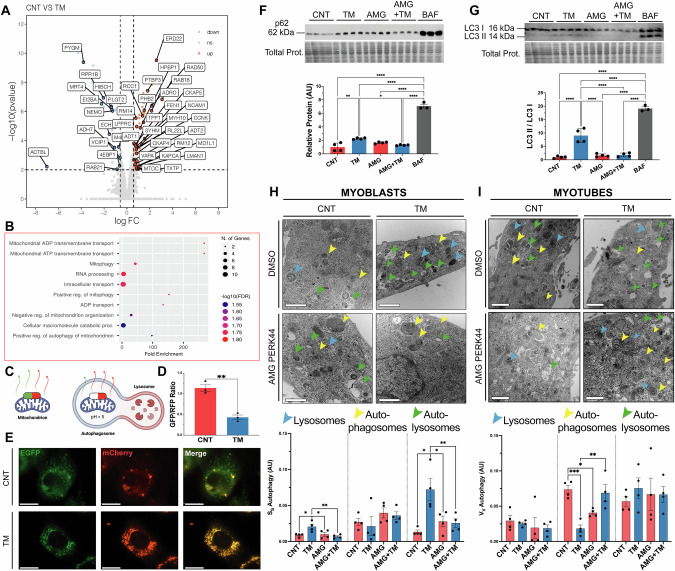
Adaptive UPR^ER^ signalling in myoblasts promotes autophagy and mitochondrial turnover. **A** Volcano plot of LFQ proteomic data of protein abundance following TM treatment (FDR = 1.58%). The cut-offs (represented as dotted lines) for significant changes are FC > 1.5 and P < 0.01. Blue dots indicate down-regulated proteins, red dots indicate up-regulated proteins. Four biological replicates per condition. **B** GOBP ShinyGO enrichment of significantly upregulated proteins. **C** Graphical diagram of the pH-sensitive construct for visualisation of mitochondrial turnover. **D**, **E** Increased mitophagy in TM treated myoblasts, represented by a decrease in the GFP/RFP ratio.; n = 3. Mean ± SEM; **p ≤ 0.01 Student’s *t* test. Scale 25 μm. **F**, **G** Western blot of protein extracts for p62 and LC3 II/I. Each lane represents independent biological replicates, data represented as mean ± SEM; *p ≤ 0.05, **p ≤ 0.01 and ***p ≤ 0.001 One-way ANOVA. **H** Representative TEM images of sections of TM treated myoblasts and quantification of the lysosome, autophagasomes and autolysosome fractions. n = 4. Scale 400 nm. Mean ± SEM; *p ≤ 0.05 and **p ≤ 0.01 One-way ANOVA. **I** Representative TEM images of sections of myotubes treated with TM at myoblast stage and quantification of lysosome, autophagasome and autolysosome fractions. n = 4. Scale 400 nm. Mean ± SEM; *p ≤ 0.05, **p ≤ 0.01 and ***p ≤ 0.001 One-way ANOVA.

To determine the mechanisms underlying the adaptations promoted by the UPR^ER^ in myoblasts, proteomic changes induced by an 8 h treatment with 0.2 μg/ml TM was performed. Global label-free proteomics quantified 2513 proteins, 71 proteins were significantly upregulated and 27 downregulated following TM treatment (Fig. 3A). Enrichment analysis of the 71 significantly upregulated proteins identified several pathways and proteins involved in *Mitophagy* and *mitochondrial ADP/ATP transport* (ADRO, TPP1, TXTP, MTDC, SYHM, RM12, KAPCA, ADT1 and ADT2), indicating active remodelling of the mitochondrial network and mitochondrial respiration (Supplementary Fig. 2J, K). Also, upregulated were proteins related to *Cell cycle and differentiation* (PTBP3, RCC1, CCNK, MD1L1 and MYH10) (Fig. 3A, B). One of the most significantly upregulated proteins was ERD22, a member of the KDELR family highly involved in the maintenance of ER homeostasis during adaptive UPR^ER^ signalling [48]. There was a significant increase in gene expression of *Kdelr2* following TM treatment (Supplementary Fig. 2L). No significant pathway enrichment was identified among the 27 downregulated proteins, which spanned diverse biological processes, including *mitochondrial RNA processing* and *protein translation* (4EBP1, MRT4, RPR1B and EI2BA) (Fig. 3A).

To further explore mitochondrial turnover, live-cell imaging was performed using the mitophagy reporter plasmid [*Cox8-EGFP-mCherry*] [49], transfected into myoblasts before TM treatment. The reporter contains a pH-sensitive GFP and a pH-stable RFP to track mitochondrial turnover; GFP fluorescence is quenched in acidic lysosomes, while RFP remains stable, allowing an estimation of mitophagy (Fig. 3C). TM treatment significantly reduced the GFP/RFP ratio compared to the control group, indicating an induction of mitophagy (Fig. 3D, E). Western blot analysis of autophagy-related proteins revealed that ULK1 levels were elevated in the TM group compared to the CNT, AMG, and AMG + TM groups (Supplementary Fig. 2M). p62 levels increased in the AMG and AMG + TM groups compared to the CNT and TM groups, indicating reduced autophagic flux (Fig. 3F). Finally, increased expression of ATG5 (Supplementary Fig. 2N) and LC3-II/I (Fig. 3G) in the TM group indicates enhanced autophagosome formation. Ultrastructure analysis of the autophagic bodies by TEM, evaluated changes in the volume fraction of lysosomes, autophagosomes and autolysosomes from myoblasts and myotubes. Myoblasts exhibited an apparent increase in the volume fraction of lysosomes and autolysosomes in the TM group compared to CNT, AMG and AMG + TM groups (Fig. 3H). In contrast, myotube analysis indicated no change in lysosome or autolysosomes volume fractions between groups, although there was a decrease in autophagosome volume in the TM group compared to the CNT and AMG + TM groups (Fig. 3I). Changes in [Ca^2+^]_i_ showed a slight, but not significant, increase in the maximal increase of store-operated calcium entry (SOCE) from the TM-treated myoblasts compared to controls (Supplementary Fig. 2O). Additionally, ER volume was assessed using an ER-targeting plasmid [*ER (KDEL)-mNeonGreen*] to estimate the ER membrane area relative to cellular surface area, revealing a significant expansion in ER membrane area following TM treatment (Supplementary Fig. 2P). Overall, proteomic and ultrastructural analyses revealed that TM treatment induces mitophagy and enhances autophagic flux during myogenesis. These adaptations depend on PERK activity and potentially play a crucial role in regulating organelle turnover.

### Early-life induction of the UPR^ER^ extends lifespan and preserves healthspan in *C. elegans*

**Fig. 4:**
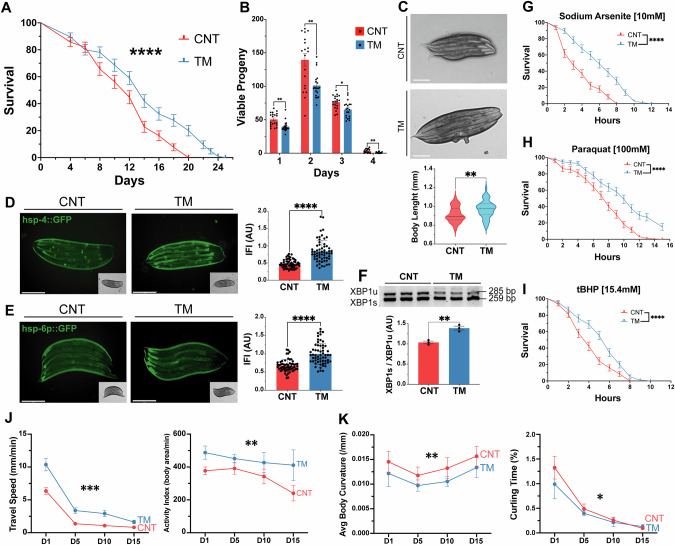
Activation of UPR^ER^ in *C. elegans* at an early developmental stage increased longevity, stress resistance and fitness. **A** Lifespan assay of TM treated N2 strain. Kaplan–Meier survival plots of two independent experiments initiated with 100 animals/group. Mean ± SEM; ****p ≤ 0.0001 Log-rank (Mantel-Cox) test. **B** TM treatment decreased progeny in N2 wild-type *C. elegans*. Data from 20 animals/group. Mean ± SEM; * p ≤ 0.05 and **p ≤ 0.01 Student’s *t* test. **C** Body length of N2 wild-type TM-treated worms. Data from 20 animals/assay. Mean ±  SEM; **p ≤ 0.01 Student’s *t* test. **D**, **E** Activation of UPR assessed by *hsp-4*::GFP (UPR^ER^)and *hsp-6p*::GFP (UPR^mt^) following TM treatment. Scale 275 μm. Mean ± SEM of at least 20 animals/assay. ****p ≤ 0.0001 Student’s *t* test. **F** Semiquantitative PCR analysis of expression ratio between XBP1s:XBP1u transcripts. n = 3, Each lane represents independent biological replicates, data represented as mean SEM; **p ≤ 0.01 Student’s *t* test. **G**, **H**, **I** Survival of wild type in Sodium Arsenite, tBuOOH and Paraquat. Kaplan–Meier survival plots of two independent experiments initiated with 45 animals/group. Mean ± SEM; ****p ≤ 0.0001 by Log-rank (Mantel-Cox) test compared with wild-type control. **J** CeleST physical fitness parameters at days 1, 5, 10, and 15. Data mean of at least 30 animals/assay. **K** CeleST physical frailty parameters at days 1, 5, 10, and 15 individual values controls and TM-treated worms during ageing. Data mean of at least 30 animals/assay.

*C. elegans* was used as a physiological model to assess the role of UPR^ER^ signalling in a whole organism. To replicate the in vitro myogenesis model, worms were treated at an early developmental stage. Following bleaching, embryos were harvested and exposed to 1.25 μg/ml TM in S Medium for 24 h and larvae plated and allowed to develop normally (Supplementary Fig. 1B) [50]. There was an increase in the longevity of TM-treated worms compared to controls (Fig. 4A). To determine if the response was an ER stress response or specific to TM, the SERCA inhibitor TG was used. Treatment with 150 nM of TG also promoted an increase in lifespan and increased filamentous mitochondrial network (Supplementary Fig. 3A, B). Reproductive potential was significantly reduced in the TM group (Fig. 4B). Importantly, this decrease in progeny was not associated with increased embryo lethality (Supplementary Fig. 3C). Interestingly, TM-treated worms exhibited increased body length compared to controls (Fig. 4C). The activation of both the UPR^ER^ and the UPR^mt^ was confirmed using transcriptional GFP reporter strains for *hsp-4* (orthologue of mammalian HSPA5/Grp78) and *hsp-6p* (ortholog of the human mtHSP70). TM treatment resulted in greater UPR^ER^ and UPR^mt^ activation capacity compared to controls (Fig. 4D, E). Analysis of the IRE-1 branch of the UPR revealed increased splicing of XBP-1 in TM-treated worms (Fig. 4F). The survival of the worms against a range of different toxicants was assessed. The resistance of the TM treated group against sodium arsenite, the redox cycler Paraquat and the organic peroxide tBuOOH was higher than the control group at adult day 1 (Fig. 4G–I). CeleST (*C. elegans* Swim Test) was performed to assess physical fitness and mobility during ageing [42]. The fitness levels were evaluated on days 1, 5, 10 and 15 of adulthood. Fitness markers including wave initiation rate, travel speed and activity index decreased with age in both groups but remained significantly higher in TM-treated worms compared to controls at all time points (Fig. 4J, Supplementary Fig. 3D, E). Conversely, frailty markers including stretch, average body curvature and curling increased with age in both groups, but these were consistently lower in the TM group, indicating better maintenance of physical health in the TM-treated groups (Fig. 4K, Supplementary Fig. 3F, G). The results demonstrate that early-life UPR^ER^ activation extends lifespan, increases stress resistance and promotes healthspan by preserving physiological fitness and reducing frailty during ageing.

### Early-life induction of the UPR promotes lifespan extension in *C. elegans* through modulation of lipid metabolism and lysosome activation

**Fig. 5:**
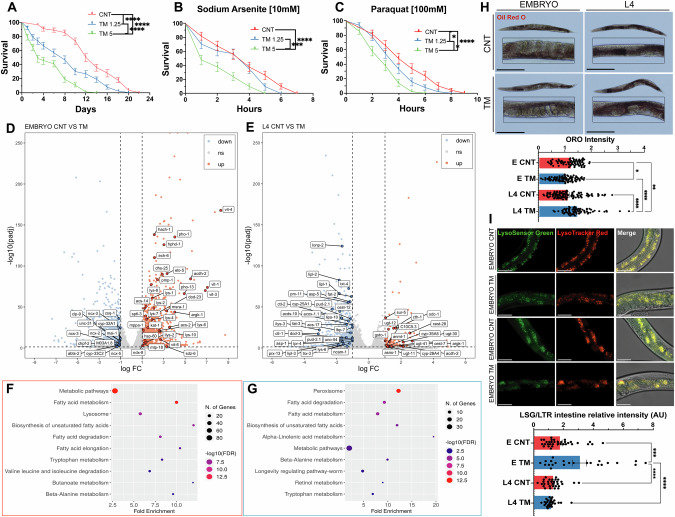
UPR^ER^ activation in *C. elegans* embryos or at L4 stage has opposite effects on lipid metabolism*.* **A** Lifespan analysis of N2 strain treated with TM at L4 stage. Kaplan–Meier survival plots of two independent experiments initiated with 100 animals. Mean ± SEM; **** p ≤ 0.0001 Log-rank (Mantel-Cox). **B**, **C** Decreased survival of N2 worms treated with TM at L4 stage in 10 mM sodium arsenite and 100 mM Paraquat. Kaplan–Meier survival plots of two independent experiments initiated with 50 animals/group. Mean ± SEM; *p ≤ 0.05, ***p ≤ 0.001 and ****p ≤ 0.0001 Log-rank (Mantel-Cox). Volcano plot of differentially expressed genes (DEGs) following TM treatment of embryos (**D**) or adults (**E**), red significantly upregulated genes and blue significantly downregulated genes. Four biological replicates per condition. **F** Dot plot of Shiny GO KEGG analysis of DEGs in embryo group. Upregulated pathways are in red and downregulated pathways are in blue. **G** Dot plot of Shiny GO KEGG analysis of DEGs in L4 group. Upregulated pathways are in red and downregulated pathways are in blue. **H** Oil Red O stained N2 worms following TM treatment at embryo or L4 stage. 3 independent experiments with at least 20 animals per strain; Scale 275 μm. Mean ± SEM; *p ≤ 0.05, **p ≤ 0.01 and ****p ≤ 0.0001 by ANOVA. **I** Intestine of wild-type worms incubated with LysoSensor Green (LSG) indicator of lysosomal pH and LysoTracker Red (LSR) indicator of lysosome content, three independent experiments with 10 animals per condition; Scale 50 μm. Mean ± SEM; ***p ≤ 0.001 and ****p ≤ 0.0001 by ANOVA.

As TM treatment of myoblasts resulted in improved myogenesis, while treatment of myotubes was detrimental, we assessed whether the adaptations promoted by the UPR^ER^ during early nematode development were similar to TM treatment of adult worms. TM treatment (1.25 and 5 μg/ml for 24 h) was performed during L4 to adult Day1 developmental stage of *C. elegans*. Both TM treatments (1.25 and 5 μg/ml) at L4 stage resulted in a significant reduction in lifespan (Fig. 5A), along with decreased resistance to paraquat and sodium arsenite (Fig. 5B, C). Interestingly, no change in resistance to sodium arsenite was observed in worms treated with the lower TM dose (1.25 μg/ml) (Fig. 5B). This highlights that the beneficial effects of TM treatment occurred only at an early developmental stage and treatment of adult worms resulted in negative effects similar to the myogenesis model.

RNA sequencing was performed to determine the molecular mechanisms underlying the different responses dependent on the developmental stage. A transcriptome analysis was conducted on Day 1 adults that were treated with TM 1.25 μg/ml at embryonic stage (EMBRYO group) (Fig. 5D) or treatment with TM at L4 stage (L4 group) (Fig. 5E). Differentially expressed genes (DEGs) were analysed (Log_2_-fold change >1 and adjusted p-value < 0.05) from EMBRYO (FDR = 2.38%) and L4 separately (FDR = 1.1%). DEGs from EMBRYO-treated groups reported 1249 upregulated and 2042 downregulated genes. Functional enrichment analysis of the upregulated genes identified pathways related to lipid metabolism and lysosome activation (Fig. 5F). Both pathways have previously been described as key intermediates in the UPR regulation of ageing [51]. Gene set enrichment analysis (GSEA) indicated enhanced expression of genes associated with autophagy (lysosomal pathways) and mitochondrial activity (oxidative phosphorylation) (Supplementary Fig. 3I). Lipid biosynthesis emerged as the most prominent upregulated pathway and there was an increase in most intermediates of this pathway (Supplementary Fig. 4A). Gene set enrichment analysis (GSEA) indicated positive enrichment of genes associated with autophagy (lysosomal pathways) and mitochondrial activity (oxidative phosphorylation) (Supplementary Fig. 4B). In contrast, the L4 group resulted in 146 upregulated and 811 downregulated genes (Fig. 5F). Functional enrichment of the downregulated genes highlighted pathways involved in lipid metabolism and *C. elegans* longevity (Fig. 5G). Pathway enrichment analysis identified a reduction in the expression of intermediates in the lipid biosynthesis pathway in the L4 treated group (Supplementary Fig. 4D). GSEA revealed decreased expression of genes related to autophagy (lysosomes) and mitophagy in L4-treated worms (Supplementary Fig. 4E). These results were validated by qPCR of genes involved in lysosomal function (*vha-6*, *asah-1* and *cpr-1*) and lipid metabolism (*acox-1.5*, *elo-2* and *fat-5*), increased expression in the EMBRYO treated group and decreased expression of fat-5 and vha-6 in the L4 treated groups (Supplementary Fig. 4G, H). Finally, comparing the functional enrichment of downregulated genes in the EMBRYO group with the upregulated genes in the L4 group demonstrated shared pathways related to metabolism (Supplementary Fig. 3C–F). These findings suggest that UPR^ER^ activation during early development promotes beneficial adaptations that contribute to lifespan extension, whereas induction later in life leads to a decline in similar protective mechanisms, particularly affecting lipid metabolism and autophagy.

RNA sequencing highlighted the effects of TM treatment on lipid metabolism and the lysosome. Oil Red O staining, which stains neutral triglycerides and lipids, was performed in the different groups [40]. Interestingly, TM treatment decreased lipid staining in the EMBRYO group compared to the controls (Fig. 5J). However, in the L4 group, TM treatment increased lipid staining compared to the control group (Fig. 5J), supporting the transcriptomic data of increased expression of lipid-related genes in embryo-treated worms but a decrease in L4 treated worms. The lysosomal content and acidity were estimated using the LysoSensor green/LysoTracker red (LSG/LSR) ratio in the intestine [52]. Results demonstrated decreased pH in the lysosomes of N2 worms subjected to the TM treatment at the embryonic stage. No changes in lysosomal pH were reported in the animals treated at the L4 stage (Fig. 5K). An upregulation of lysosomal genes and increased intestinal lysosomal acidity in response to UPR^ER^ activation has previously been demonstrated to improve proteostasis and longevity in *C. elegans* [53]. RNAi knockdown of *lmp-1* (ortholog of LAMP1, a lysosomal membrane protein important for lysosomal function) had no effect on longevity or in combination with TM treatment (Supplementary Fig. 3L). RNAi knockdown of *lmp-1* also resulted in a more fragmented mitochondrial network after TM treatment (Supplementary Fig. 3M). These results suggest activation of autophagy is required for the adaptive UPR^ER^ increase in longevity and mitochondrial morphology. Overall, the effects of TM treatment were dependent on the developmental stage and resulted in the opposite expression of genes involved in lipid metabolism, and subsequent effects on longevity and oxidative stress resistance. The results support the findings in the myogenesis model and highlight increased plasticity for adaptations following UPR^ER^ induction at early developmental stages.

### Early-life UPR induction stimulates MERCS formation, mitochondrial capacity and mitophagy in *C. elegans*

**Fig. 6:**
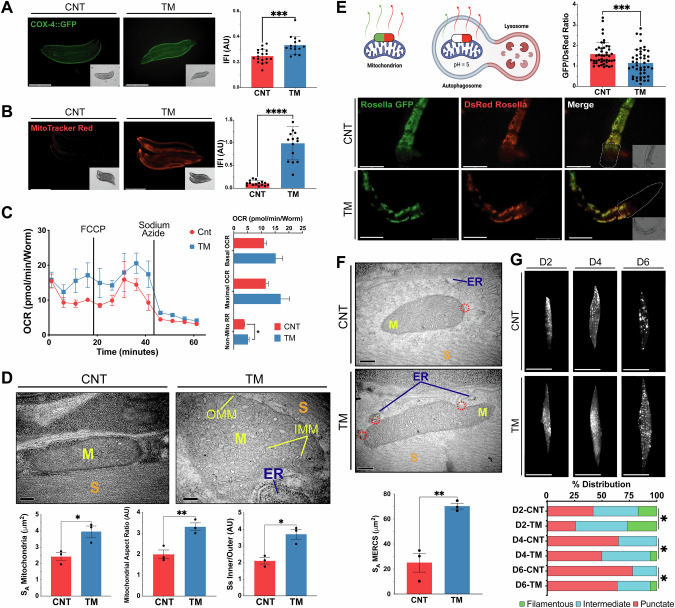
Early-life exposure of *C. elegans* to ER stress improves mitochondrial capacity, activates mitophagy and promotes MERCS assembly. **A** Mitochondrial content assessed using transgenic day 1 worms *pcox-4*::*gfp* following TM treatment. Three independent experiments with 15 animals/group. Scale 275 μm. Mean ± SEM; ***p ≤ 0.001. Student’s *t* test. **B** MitoTracker red staining of N2 strain following TM treatment. Three independent experiments were initiated with 15 animals/group. Scale 275 μm. Mean ± SEM; ****p ≤ 0.0001. Student’s *t* test. **C** Oxygen consumption of adult day 1 N2 worms following treatment with TM. Data from 3 independent experiments with 8–12 animals per well. Mean ± SEM; * p < 0.05 Student’s *t* test. **D** Representative TEM images of sections from day 1 *C. elegans* body wall muscle cells. Mitochondrial surface area, aspect ratio and IMM/OMM surface area ratio were calculated. Scale 200 nm. Mean ± SEM; *p ≤ 0.05 and **p ≤ 0.01 Student’s *t* test. **E** Representative images of the head region of the *pmyo-3::TOMM-20::Rosella* day 1 reporter strain following TM treatment from 3 independent experiments initiated with 15 animals/group; **p < 0.01 Student’s *t* test. Scale 75 μm. **F** Representative TEM images of sections from day 1 *C. elegans* body wall muscle cells. Representative images of 3 independent experiments initiated with 10 animals/group. Scale 200 nm. Mean ± SEM; **p ≤ 0.01 Student’s *t* test. **G** Representative images of day 2, 4 and 6 p*myo-3::mitogfp* reporter strain for muscle mitochondrial morphology, classified as either filamentous, intermediate or punctate. Three independent experiments were initiated with 15 animals/condition. Scale 50 μm. n = 3 *p < 0.05 Chi-square.

In order to determine if there was a similar adaptive response in nematodes to the myogenesis model following TM treatment, the effects on mitochondrial content were assessed using a transcriptional P*cox-4*::GFP reporter [54]. The fluorescence intensity increased with TM treatment (Fig. 6A). MitoTracker Red staining was used to estimate mitochondrial membrane potential and there was a significant increase in the fluorescence intensity following treatment with TM (Fig. 6B), which was also observed using TMRM staining (Supplementary Fig. 3N). In addition, mitochondrial superoxide production was evaluated by MitoSOX staining, demonstrated a reduction in the TM group (Supplementary Fig. 3O). Mitochondrial respiration was assessed using adult day 1 *C. elegans* following treatment with TM at the embryo stage. TM-treated worms showed a slight, non-significant increase in both basal and maximal OCR, and there was a significant increase in non-mitochondrial respiration (Fig. 6C). TEM was used to analyse the mitochondrial ultrastructure of body wall muscle cells from *C. elegans* (Supplementary Fig. 1D). Stereological analysis revealed increased mitochondrial surface area in the TM group (Fig. 6D). Moreover, mitochondria from the TM treatment group, exhibited an increased aspect ratio, indicating more elongated, fused mitochondria. The surface area ratio between the inner (IMM) and outer mitochondrial membranes (OMM) was also significantly increased, reflecting a denser IMM structure (Fig. 6D), which may contribute to the observed improvements in mitochondrial function.

Mitochondrial turnover was assessed using the *pmyo-3::TOMM-20::Rosella* reporter strain, containing a pH-sensitive Rosella biosensor composed of a pH-sensitive GFP and a pH-stable RFP to track mitochondrial turnover in body wall muscle [55]. TM-treated worms exhibited a significantly lower GFP/DsRed fluorescence ratio, indicating increased mitophagy and enhanced mitochondrial turnover during adulthood (Fig. 6E). RNAi knockdown of *pdr-1* (ortholog of Parkin, regulator of mitochondrial turnover) had no effect on longevity but in combination with TM treatment decreased lifespan compared with TM alone, RNAi knockdown of *pdr-1* resulted in a more fragmented mitochondrial network (Supplementary Fig. 3J, K). RNAi knockdown of *lgg-1* (ortholog of LC3, required for autophagosome maturation) following TM treatment resulted in no change in lifespan or on mitochondrial morphology (Supplementary Fig. 3H, I). These results indicate activation of mitophagy is required for the increased longevity and changes in mitochondrial morphology following adaptive UPR^ER^ signalling.

As MERCS are essential for mitochondrial fission, TEM analysis was performed to quantify the surface area of mitochondria in close contact with the ER (<50 nm). TM-treated worms resulted in a marked increase in MERCS surface area compared to controls (Fig. 6F). Mitochondrial morphology was evaluated during ageing using a genetic p*myo-3::mitoGFP* reporter, for visualisation of the mitochondrial network from body wall muscle. Images were taken on days 2, 4 and 6 of adulthood and classified as punctate, intermediate or filamentous, depending on the mitochondrial network morphology [56]. Ageing resulted in a progressive shift towards more punctate (fragmented) networks (Fig. 6G). In contrast, TM-treated worms demonstrated a delayed onset of mitochondrial fragmentation, maintaining more filamentous and intermediate networks at all time points assessed (Fig. 6G). In order to investigate whether this response is redox dependent, the antioxidant N-Acetyl Cysteine (NAC) was used. TM treatment during early development in combination with NAC resulted in the repression of lifespan extension and increased mitochondrial fragmentation, indicating the beneficial adaptive UPR response has a redox component (Supplementary Fig. 3P, Q). Together, the data demonstrate that, similar to myoblasts, early-life TM treatment helps preserve mitochondrial network integrity and morphology during ageing, potentially through the formation of MERCS and regulation of mitochondrial turnover.

### PEK-1 (UPR^ER^) and ATFS-1 (UPR^mt^) crosstalk regulates the adaptations promoted by the UPR in *C. elegans*

**Fig. 7:**
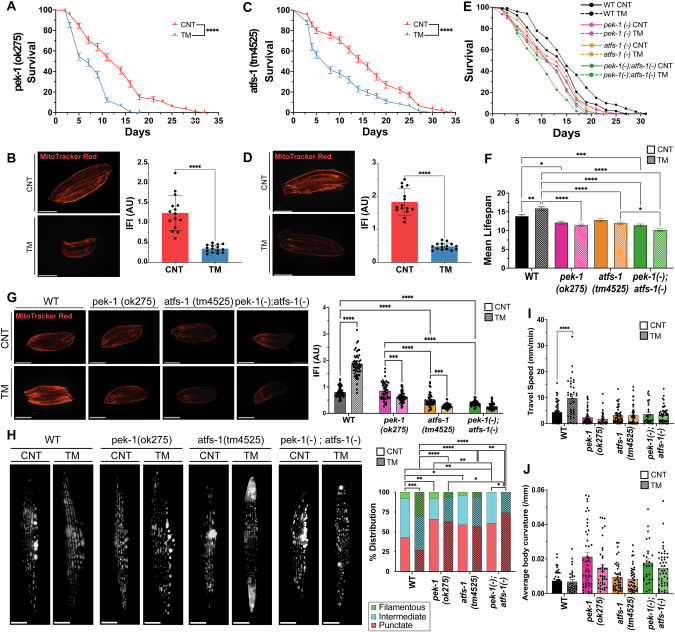
Adaptations to early-life adaptive UPR^ER^ in *C. elegans* depend on PEK-1 and ATFS-1 crosstalk. **A** Lifespan analysis of *pek-1*(ok275) strain following treatment with TM. Kaplan–Meier survival plots of two independent experiments initiated with 100 animals/group, ****p ≤ 0.0001 Log-rank (Mantel-Cox) test. **B** MitoTracker Red images of TM-treated day 1 *pek-1*(ok275) worms. Scale 275 μm. Mean ± SEM; ****p ≤ 0.0001 Student’s *t* test. **C** Lifespan analysis of *atfs-1*(tm4525) following treatment with TM. Kaplan–Meier survival plots of two independent experiments initiated with 100 animals/group, ****p ≤ 0.0001 Log-rank (Mantel-Cox) test. **D** MitoTracker Red images of TM-treated adult day 1 *atfs-1*(tm4525) worms. Scale 275 μm. Mean ± SEM; **** p ≤ 0.0001 Student’s *t* test. **E** Lifespan assay of N2 Wild-type, *pek-1*(ok275), *atfs-1*(tm4525) and *pek-1*;*atfs-1* worms following TM treatment Kaplan–Meier survival plots of two independent experiments initiated with 100 animals per group. **F** Mean lifespan of N2, *pek-1*(ok275), *atfs-1*(tm4525) and *pek-1*;*atfs-1* strains. *p ≤ 0.05, **p ≤ 0.01, ***p ≤ 0.001, ****p ≤ 0.0001 One-way ANOVA. **G** MitoTracker Red images of day1 N2, *pek-1*(ok275), *atfs-1* (tm4525) and *pek-1*;*atfs-1* strains. Data mean SEM of 45 animals/assay. *** p ≤ 0.001, ****p ≤ 0.0001 One-way ANOVA. **H** Images of p*myo-3*::*mitogfp* reporter strain adult day4 N2 WT, *pek-1*(ok275), *atfs-1*(tm4525) and *pek-1*;*atfs-1* backgrounds. 3 independent experiments with 15 animals/condition, n = 3; Scale 12 μm. *p ≤ 0.05, **p ≤ 0.01, ***p ≤ 0.001, ****p ≤ 0.0001 Chi-square. **I** CeleST physical fitness parameters at day 1 following TM treatment of N2 WT, *pek-1*, *atfs-1* and *pek-1*;*atfs-1* strains. Data from 3 independent experiments initiated with at least 10 animals per condition. Mean ± SEM; **p ≤ 0.01, ***p ≤ 0.001 One-way ANOVA. **J** CeleST physical fitness parameters that increase with age; average fitness parameters at day 1 following TM treatment of N2 WT, *pek-1*, *atfs-1* and *pek-1*;*atfs-1* mutant strains. Data from three independent experiments initiated with at least 10 animals/conditions. Mean ± SEM; **p ≤ 0.01 and ****p ≤ 0.0001 One-way ANOVA.

To elucidate the mechanisms underlying UPR-mediated mitochondrial and physiological adaptations, we used mutant strains for the UPR^ER^ and UPR^mt^ signalling arms to evaluate their response to early-life TM treatment. Both the *ire-1(v33)* and *xbp-1(tm2482)* mutant strains were viable. However, TM treatment resulted in developmental arrest at the larval stage (Supplementary Fig. 3A). This phenotype has previously been reported, generation of double mutants of *ire-1* or *xbp-1* with *atf-6* or *pek-1* promoted developmental arrest at larvae stage L2 [57]. highlighting the critical role of the IRE-1 arm in the resistance to ER stress. Similar to the N2 strain, treatment of the *atf-6 (ok551)* mutant strain with TM resulted in increased longevity and stress resistance to sodium arsenite but not paraquat but increased MitoTracker Red staining (Supplementary Fig. 5B). Interestingly, following TM treatment, *atf-6* mutants exhibited a reduction in size at day 1 of adulthood, implying delayed development (Supplementary Fig. 5B). As the increased longevity and survival following TM treatment were also observed in N2 strain (Fig. 4A, G, H), it would suggest they are independent of ATF-6 signalling. Following TM treatment of the PEK-1 loss-of-function mutant strain (*pek-1* (ok275)), there was a decrease in lifespan (Fig. 7A), resistance to paraquat was reduced but resistance to sodium arsenite was unaltered (Supplementary Fig. 5C). There was a decrease in MitoTracker Red fluorescence intensity in the TM-treated *pek-1* mutants (Fig. 7B). The *pek-1* mutant strain also had a reduction in size following TM treatment (Supplementary Fig. 5C). TM treatment of *pek-1* mutant strain produced an opposite phenotype compared to the N2 strain, highlighting its importance for this adaptive response. TM treatment of ATFS-1 mutant (*atfs-1* (tm4525)) also exhibited a decrease in lifespan (Fig. 7C); however, resistance to oxidative stressors was unaltered (Supplementary Fig. 5D). The ΔΨm of the TM-treated *atfs-1* mutant strain decreased following TM treatment (Fig. 7D). Interestingly, TM-treated *atfs-1* mutant exhibited no differences in size compared to the control group (Supplementary Fig. 5D). The results highlight that TM treatment of the *atfs-1* mutant strain had similar results to the *pek-1* mutant and an opposite phenotype to N2 WT strain, highlighting its importance for this adaptive response.

A double *pek-1*;*atfs-1* mutant was generated to clarify the possible interaction of these signalling pathways and potential crosstalk of the UPR^ER^ and UPR^mt^. The phenotypic effects of TM treatment in the N2 wild-type strain, *pek-1*, *atfs-1* and *pek-1*;*atfs-1* strains were determined. All mutant strains had decreased lifespan compared to the WT group which had increased longevity (Fig. 7E, F, Supplementary Table 1). Interestingly, TM increased the size of N2 worms, but this effect was reversed in the *pek-1* mutant and absent in the *atfs-1* mutant (Supplementary Fig. 5E). The *pek-1;atfs-1* double mutant exhibited a more severe reduction in body size than either single mutant, both under basal conditions and following TM treatment (Supplementary Fig. 5E). All mutant strains treated with TM had decreased resistance to sodium arsenite and paraquat compared to N2 strain (Supplementary Fig. 5E, Supplementary Tables 2, 3).

Mitochondrial membrane potential was significantly reduced in both the *atfs-1* and *pek-1;atfs-1* mutants compared to N2, while no differences were observed between the untreated N2 and the *pek-1* mutant strain (Fig. 7G). TM treatment only increased mitochondrial membrane potential in N2 WT worms, with all mutants displaying lower MitoTracker Red staining post-TM treatment (Fig. 7G). To assess mitochondrial morphology in body wall muscle, strains were crossed with the p*myo-3*::mitoGFP reporter strain and imaged at day 4 of adulthood. Control N2 WT worms had a balanced mitochondrial network with approximately 20% filamentous, 40% intermediate, and 40% punctate mitochondria. The *pek-1* and *atfs-1* mutants demonstrated increased punctate or more fragmented mitochondrial network (Fig. 7H). However, the *pek-1;atfs-1* double mutant displayed a highly disrupted mitochondrial network, with no filamentous mitochondria present. TM treatment increased the percentage of filamentous mitochondria in N2 WT worms (~25%) and reduced the percentage of mitochondrial punctate (~25%). In contrast, the mitochondrial morphology of *pek-1* and *atfs-1* single mutants remained unchanged following TM treatment, while the *pek-1;atfs-1* mutant exhibited further mitochondrial fragmentation (Fig. 7H). Finally, to assess physiological activity, CeLeST analysis was performed. In untreated conditions, fitness-related parameters were similar between WT, *pek-1*, *atfs-1*, and *pek-1;atfs-1* mutants, while frailty parameters were slightly higher in the mutant strains (Fig. 7I, J, Supplementary Fig. 3F). Following TM treatment, WT worms had increased wave initiation rate, travel speed and activity index indicating increased fitness, this was not observed in any of the mutant strains (Fig. 7, Supplementary Fig. 3F). To dissect the mechanistic basis of this crosstalk we evaluated the cellular response to early-life ER stress. Downstream markers of the UPR^ER^ and UPR^mt^ (*hsp-4*, a canonical UPR^ER^ target, and *hsp-6*, a mitochondrial chaperone and target of ATFS-1) exhibited a clear increase in both following TM treatment in the N2 strain. However, mutant strains *pek-1*(-), *atfs-1*(-) and *pek-1*(-);*atfs-1*(-) did not increase *hsp-4* or *hsp-6* following TM treatment suggesting both PEK-1 and ATFS-1 are required for adaptive UPR activation (Supplementary Fig. 5G).

Together, these results highlight the critical roles of PEK-1 and ATFS-1 in regulating lifespan, stress resistance and mitochondrial dynamics. The results indicate that under mild ER stress conditions, PEK-1 from the UPR^ER^ establishes crosstalk with ATFS-1 from the UPR^mt^ pathway to promote increased lifespan, improved healthspan, enhanced stress resistance and greater mitochondrial function. Moreover, based on the gene expression profile of UPR markers, the slight increase in these markers with the TM treatment elicit an adaptive UPR activation, whereas the more drastic activation of the markers in the AMG + TM group could indicate an induction of maladaptive UPR signalling.

In summary, the data presented using both an in vitro myogenesis model and in vivo whole organism *C. elegans*, reveals that adaptive UPR^ER^ signalling through PERK and UPR^mt^, induces the assembly of MERCS and regulates mitochondrial adaptations following a low dose of an ER stressor at an early developmental stage. These adaptations promote myogenesis and extension of lifespan in *C. elegans* by enhancing organelle turnover and mitochondrial function, demonstrating the essential crosstalk between ER and mitochondrial stress responses in maintaining cellular and organismal homeostasis under physiological stress conditions.

## Discussion

This study identified a key role of adaptive UPR^ER^ signalling during early development, which regulates the assembly of MERCS, mitochondrial function, and subsequent physiological outcomes. Acute exposure to a low level of the ER stressor, TM, induced adaptive UPR^ER^ signalling and promoted adaptations in both in vitro and in vivo models. The adaptative responses, including increased mitochondrial content and activity, depend on the PERK arm of the UPR^ER^. A low concentration of TM enhanced myogenic potential in C2C12 myoblasts, while higher concentrations induced cell death. We propose that the improved myogenesis is a result of increased MERCS assembly and increased mitochondrial turnover and content. Similarly, in *C. elegans*, UPR^ER^ activation at an early developmental stage resulted in significant lifespan extension and stress resistance during ageing. TM treatment promoted MERCS assembly, mitochondrial turnover and increased lipid metabolism in *C. elegans*. Previous studies in *C*. *elegans* have reported that an ER stress response can extend lifespan [50]. Indeed, expression of constitutively active neuronal XBP-1s can activate UPR^ER^ in distal tissues resulting in increased longevity [58], modulating lipid metabolism and increased lysosomal acidity [53, 59]. Mechanistically, the results presented here identified PEK-1 (PERK) as the essential regulator of adaptive UPR^ER^ signalling and determined crucial crosstalk between PEK-1 (UPR^ER^) and ATFS-1 (UPR^mt^) in maintaining mitochondrial integrity and promoting longevity. Although TM treatment increased longevity and preserved mitochondrial morphology, it was associated with decreased reproductive potential, supporting previous studies that UPR^ER^ induction promotes the allocation of organismal resources toward the maintenance of somatic cells and decreases reproductive potential [60].

In both in vitro and in vivo models, TM treatment increased mitochondrial content, turnover and respiration. Disruption of the ER environment has been associated with downstream effects on mitochondrial function [13]. The ER can coordinate mitochondrial dynamics by establishing contact sites through ER tubules [19]. INF2 from the ER interacts with the OMM actin nucleator Spire1c to polymerise actin filaments around mitochondria [61], stimulating ER tubules to release Ca^2+^ ions into mitochondria through VDAC1, triggering the inner mitochondrial membrane to divide [62]. MERCS also mediate the replication and distribution of mtDNA along the mitochondrial network during mitochondrial fission [63, 64]. ER tubules can guide the position and timing of mitochondria fusion through tethering with mitochondria [65], as MERCS must be maintained to decrease mitochondrial motility [66]. Narrow MERCS (<10 nm) are characterised by an efficient transfer of molecules and signals, which enables a rapid response to metabolic changes and stress signals [67]. Contact sites facilitate the efficient and rapid transfer of Ca^2+^ ions from the ER to mitochondria, crucial for maintaining cellular Ca^2+^ homeostasis and mitochondrial function [68]. MERCS also allow an efficient exchange of lipids between the ER and mitochondria, essential for membrane biosynthesis and the maintenance of mitochondrial structure [69]. Long-distance MERCS (>30 nm) limit the extent and speed of interactions, ensuring that only necessary signalling occurs [67]. It has been reported that long-distance MERCS prevent excessive build-up of Ca^2+^ within the mitochondria, which can result in the opening of the mPTP, mitochondrial dysfunction and initiation of apoptotic signalling events [68].

The presence of ROS within MERCS has been reported to generate redox nanodomains between the two organelles, which have been proposed to mediate redox signalling and IP3R-mediated Ca^2+^ release via MERCS, resulting in the swelling of the mitochondrial matrix, reduction of the cristae and release of H_2_O_2_ [70]. TEM analysis demonstrated an increase in MERCS following TM treatment at the embryonic stage which was maintained following differentiation of myoblasts into myotubes and in adult worms. Moreover, addition of the antioxidant NAC alongside the TM treatment prevented the beneficial lifespan and mitochondrial morphology adaptations, suggesting a potential redox component in the signalling required for the adaptive response and MERCS assembly. The PERK arm of the UPR^ER^ can modulate mitochondrial capacity in response to acute ER stress, promoting the remodelling of the mitochondrial network by induction of SIMH [13, 14]. Adaptive UPR^ER^ signalling can induce the formation of MERCS, promoting Ca^2+^ transfer between these organelles and boosting mitochondrial metabolism by increasing the activity of the ETC and enzymes of the TCA cycle [21]. PERK localises to mitochondrial-associated membranes (MAMs) and its interaction with the mitochondrial tethering protein MFN2 stabilises MERCS [71]. Ablation of PERK disrupts ER morphology and reduces the number of MERCS, indicating that PERK is required at the ER for tethering mitochondrial membranes [47]. How PERK regulates MERCS assembly is still uncertain. It has been demonstrated that cells lacking PERK have disrupted MERCS assembly [2, 47]. However, rescuing cells lacking PERK with kinase-dead PERK mutants restored MERCS assembly, indicating its kinase activity is not essential for maintaining MERCS [2, 47]. The stabilisation of MERCS depends on PERK’s cytosolic domain, as overexpression of PERK lacking this domain did not restore MERCS assembly, suggesting that PERK’s tethering role is structural rather than dependent on canonical PERK signalling [47]. It has been suggested that the non-canonical role of PERK in regulating MERCS is via recruiting lipid transfer proteins such as Extended-Synaptotagmin-1 (E-Syt-1) [72]. PERK also possess a conserved cysteine residue (Cys216) that can be reversibly oxidised, allowing the formation of covalent interactions with ERO1α and tightening of MERCS [2].

Activation of the UPR^ER^ during the transition from L4 larvae to adult day 1 stage had opposite effects compared to those reported following treatment at the embryonic stage, decreasing both lifespan and resistance to oxidative stress. In *C. elegans*, UPR^ER^ activation in response to ER stress begins to decline relatively early in the ageing process [58, 73], coinciding with the peak of the reproductive period [74]. UPR^ER^ induction promotes the allocation of organismal resources toward the maintenance of somatic cells, thereby decreasing reproductive potential [60]. As cells age, their ability to maintain proteostasis diminishes, highlighting the importance of sustaining UPR^ER^ function to prevent age-related declines in homeostasis [60]. Transcriptomic analysis revealed that TM treatment during early development upregulated genes involved in lipid metabolism and autophagy, contributing to lifespan extension. In contrast, TM treatment of adult worms reduced lipid metabolism, impairing survival. Enhanced longevity in *C*. *elegans* is linked to the transcriptional upregulation of genes related to lysosomal function and the modulation of fatty acid desaturases, lysosomal lipases and general lipophagy [53, 59, 75]. Lipid staining confirmed these changes, with embryo TM-treated worms having lower lipid stores and increased expression of genes involved in lipid synthesis and decreased lysosomal pH, while adult TM-treated worms had higher lipid levels and decreased expression of genes involved in autophagy. Lipid biosynthesis requires phospholipid exchange between the ER and the mitochondria at MERCS. The data presented support the role of PERK in promoting lipid trafficking at MERCS [72].

To investigate the IRE-1 arm of the UPR^ER^, mutant strains for *ire-1* and *xbp-1* were used, but all larvae arrested following TM treatment. Although the mutant strains of the IRE-1 arm were viable, when faced with an additional ER stress during early development, the larvae arrested. A similar effect has been reported in *ire-1* or *xbp-1* mutant strains with the deletion of *atf-6* or *pek-1* [57]. In this study, *pek-1* mutants subjected to TM treatment exhibited a reduction in lifespan, oxidative stress resistance and mitochondrial membrane potential [57]. In myoblasts, PERK inhibition blocked the myogenic and mitochondrial adaptations. Cells deficient in PERK, exhibit disrupted ETC activity and increased mitochondrial ROS [76]. Following TM treatment the *atfs-1* mutant exhibited a similar phenotype to that of the *pek-1* mutant, both of which responded oppositely compared to the N2 wild-type strain. The observed phenotypic changes were also reduced following TM treatment in *pek-1;atfs-1* double mutants, suggesting that *pek-1* and *atfs-1* operate within the same signalling pathway. Based on the results presented, PERK can regulated mitochondrial remodelling through the assembly of MERCS, possibly via alterations in the redox environment and recruitment of lipid transfer proteins at MAMs. Activation of UPR^mt^ following ER stress aims to restore mitochondrial function and proteostasis by promoting mitochondrial repair and mitophagy to prevent mitochondrial dysfunction [77].

The data presented provides valuable insights into the role of UPR^ER^ signalling and mitochondrial dynamics during development and ageing; but does not fully address the temporal dynamics of these pathways and has a number of limitations. The results indicate a potential redox signalling mechanism in orchestrating MERCS assembly, such a ‘redox nanodomain’ at contact sites has previously been reported [70]. Tools to accurately quantify localised ROS generation and specific redox modifications on regulatory proteins would identify the mechanisms that coordinate MERCS assembly. Similarly, the distance between the ER and mitochondria in the MERCS is a key factor determining MERCS’ function. Recently, it was demonstrated that MERCS distance determines the transfer of oxidised phospholipids to mitochondria in the cellular response to ferroptosis [78]. In this study, PERK inhibition in the cell model was performed using pharmacological approaches which can be subject to non-specific effects. A complimentary genetic approach using site-specific modifications of its key regulatory sites that facilitate MERCS assembly, would identify the underlying mechanism. The use of both *C. elegans* and mammalian cell lines allows for a better understanding of the regulatory signalling mechanisms underlying MERCS assembly and communication between the ER and mitochondria.

In summary, the results highlight that activation of an adaptive UPR^ER^ response during early developmental stages promoted PERK-dependent assembly of MERCS that determines mitochondrial function and dynamics, as well as the regulation of genes related to lysosomal function and lipid metabolism. The interaction between PEK-1 (UPR^ER^) and ATFS-1 (UPR^mt^) in response to early-life ER stress was essential for maintaining mitochondrial dynamics and function, lifespan extension and stress resistance.

## Supplementary information

Supplementary material

## Data Availability

Proteomic data has been deposited through ProteomeXchange (PXD059735) and the RNA-seq data has been deposited at GEO (GSE285634), they will be publicly available as of the date of publication. Accession numbers are listed in the key resources table. Original Western blot images and microscopy data reported in this paper have been deposited at Mendeley Data 10.17632/sztmnwvsd9.1. Any additional information required to reanalyse the data reported in this paper is available from the lead contact upon request.
