# Supplementary material for: Adaptive ER stress promotes mitochondrial remodelling and longevity through PERK-dependent MERCS assembly
Source: Cell Death Differ. 2025 Nov 1;33(4):732–47. doi: 10.1038/s41418-025-01603-7 (PMC13076674; doi:10.1038/s41418-025-01603-7)
Supplement: Supplementary file 1 — Supplementary material [file 41418_2025_1603_MOESM1_ESM.docx]

**List of contents for Supplemental Information**

- **Supplementary Figures**

Supplementary Figure 1. Myoblast and *C. elegans* models for determination of the effects of adaptive UPR^ER^ signalling during early developmental stages.

Supplementary Figure 2. Effect of Tunicamycin treatment of myoblasts on myogenesis, mitochondrial and ER content and volume.

Supplementary Figure 3. Early-life induction of UPR^ER^ in *C. elegans* activates autophagy and promotes physiological adaptations.

Supplementary Figure 4. Early-life Tunicamycin treatment of *C. elegans* embryos or at L4 stage promotes opposite effects on lipid metabolism and lysosomal function.

Supplementary Figure 5. Early-life Tunicamycin treatment *of C. elegans* requires the PEK-1 arm of the UPRER for the beneficial adaptive response.

- **Supplementary Tables**

Supplementary Table 1: Multiple comparison lifespan analysis at 20°C of wild-type (WT), *pek-1*(ok275) (PEK), *atfs-1(*tm4525) (ATFS) and *pek-1;atfs-1*.

Supplementary Table 2: Multiple comparison Sodium Arsenite survival analysis at 20°C of wild-type (N2), *pek-1*(ok275) (PEK), *atfs-1*(tm4525) (ATFS) and pek-1;atfs-1.

Supplementary Table 3: Multiple comparison Paraquat survival analysis at 20°C of N2 wild-type (WT), *pek-1*(ok275) (PEK), *atfs-1(*tm4525) (ATFS) and *pek-1*;*atfs-1* strains.

Supplementary Table 4: Multiple comparison lifespan analysis at 20°C of wild-type L4440 CNT, L4440 TM, RNAi *lgg-1* CNT and RNAi *lgg-1* TM.

Supplementary Table 5: Multiple comparison lifespan analysis at 20°C of wild-type L4440 CNT, L4440 TM, RNAi *pdr-1* CNT and RNAi *pdr-1* TM.

Supplementary Table 6: Multiple comparison lifespan analysis at 20°C of wild-type L4440 CNT, L4440 TM, RNAi *lmp-1* CNT and RNAi *lmp-1* TM.

Supplementary Table 7: Multiple comparison lifespan analysis at 20°C of wild-type CNT, NAC, TM and NAC+TM.

Supplementary Table 8: Key resources table.

- **Uncropped Western blot membranes**

**Supplementary Figures**

**
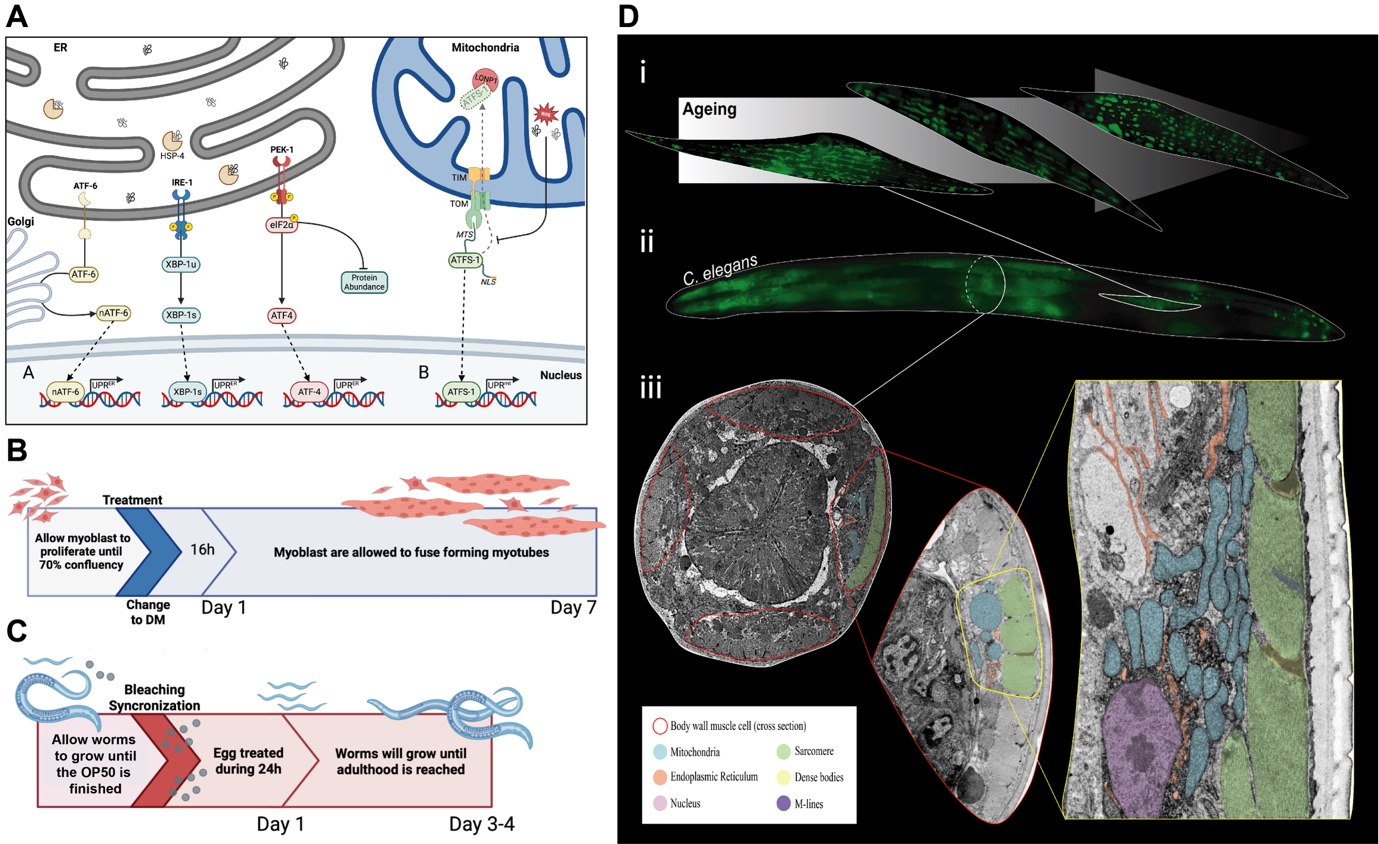
**

**Suppl. Figure 1: Myoblast and *C. elegans* models for determination of the effects of adaptive UPR^ER^ signalling during early developmental stages.**

**A)** Schematic diagram of the TM treatment protocol for C2C12 myoblasts.

**B)** Schematic diagram of the TM treatment protocol of *C. elegans* at embryo stage.

**C)** The UPR^ER^ and UPR^mt^ of *C. elegans*.

**D)** Organelle ultrastructure from the body wall muscle of *C. elegans*. (i) Muscle cell ageing visualised using a p*myo-3*::mitoGFP translational fusion highlighting the mitochondrial network in body wall cells from a young day 2, 4 & 6 adult demonstrating mitochondrial network disorganisation. (ii) The organisation of body wall muscle cells in a mature hermaphrodite *C. elegans* (seen in a dorsal oblique view) was observed in a genetically modified worm expressing the P*myo-3*::mitoGFP reporter transgene. (iii) TEM cross-section images of body wall muscle cells. The first image shows the distribution of the four muscle quadrants (containing two rows of cells in each) from the pharynx area. The second image demonstrates a cross-section of the body wall cell, in which mitochondria, ER and Sarcomeres can be distinguished. High-resolution TEM section of the nucleus, mitochondria, ER and sarcomeres (with dense bodies and M-lines) in the body wall muscle cell.

**
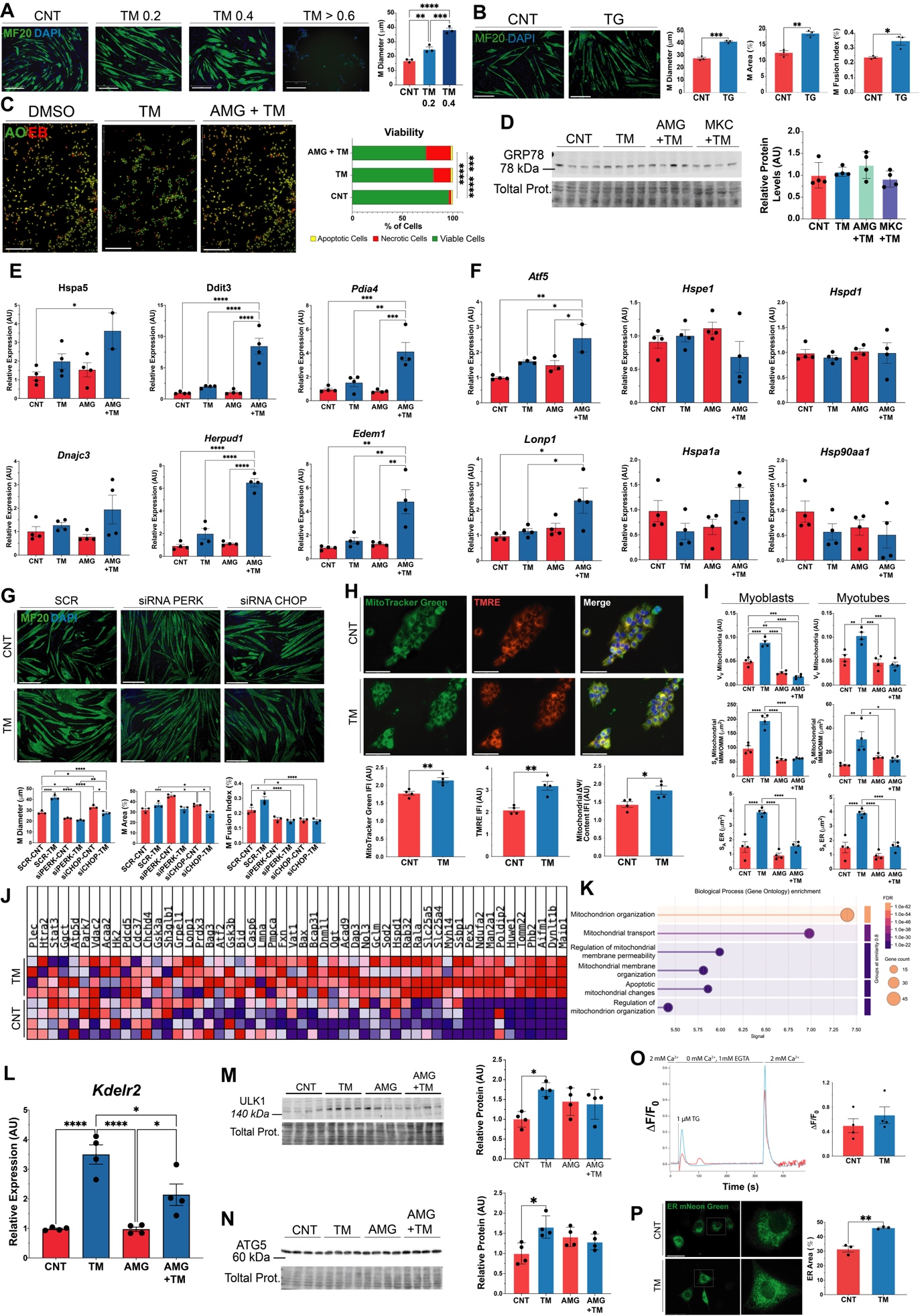
**

**Suppl. Figure 2: Effect of TM treatment of myoblasts on myogenesis, mitochondrial and ER content and volume.**

**A)** C2C12 myoblasts were exposed to TM 0.2, 0.4, 0.6, 0.8 and 1 μg/ml for 8h. Media was replaced with differentiation media and cells were allowed to differentiate for 7 days. MF20 immunostaining demonstrated that TM treatment improved the myogenic potential of the myoblast population compared to the control; this effect is lost when the concentration of TM exceeds the 0.4 μg/ml. ** p ≤ 0.01, *** p ≤ 0.001 & **** p ≤ 0.0001 One-way ANOVA.

**B)** C2C12 myoblasts were exposed to thapsigargin (TG) 10 nM for 8h. Media was replaced with differentiation media and cells were allowed to differentiate for 7 days. MF20 immunostaining demonstrated that TG treatment significantly increases myotube diameter, area and fusion index. * p ≤ 0.05, ** p ≤ 0.01 & *** p ≤ 0.001 Student’s t-test.

**C)** C2C12 myoblasts were exposed to TM 0.2μg/ml and AMG PERK44 (PERK inhibitor) 2μM for 8h. Media was replaced with differentiation media and cells were allowed to recover for 16h. Treatment with TM resulted in a significant increase in cell death compared to the control group; this effect is exacerbated with the inclusion of the PERK inhibitor. *** p ≤ 0.001 & **** p ≤ 0.0001 Chi-square.

**D)** Protein expression levels of GRP78 assessed by Western Blot, normalised against total protein (Ponceau staining). Each lane represents independent biological replicates, data represented as mean SEM. One-way ANOVA.

**E)** Gene expression levels of *Hspa5*, *Dnajc3*, *Ddit3*, *Herpud1*, *Pdia4* and *Edem1* were assessed in C2C12 myoblasts using ΔΔCT method by qPCR, and normalised against *β2 microglobulin* (B2M) housekeeper gene. * p ≤ 0.05 One-way ANOVA.

**F)** Gene expression levels of *Atf5*, *Lonp1*, *Hspe1*, *Hspa1a*, *Hspd1* and *Hsp90aa1* were assessed in C2C12 myoblasts using ΔΔCT method by qPCR, and normalised against *β2 microglobulin* (B2M) housekeeper gene. * p ≤ 0.05 One-way ANOVA.

**G)** C2C12 myoblasts were exposed to TM 0.2 μg/ml or, alongside Scr, siRNA for PERK or siRNA for CHOP during 8h. Media was replaced with differentiation media, and cells allowed to differentiate for 7 days. MF20 immunostaining demonstrated that the TM treatment increased the myogenic potential of the myoblast population compared to the control, but this effect is lost with the silencing of RNA for PERK and CHOP. * p ≤ 0.05, ** p ≤ 0.01, *** p ≤ 0.001 & **** p ≤ 0.0001 One-way ANOVA.

**H)** TM treatment resulted in a significant increase in MitoTracker green fluorescence intensity. TM treatment increased TMRE staining, indicating an increase in mitochondrial membrane potential. The TMRE:MitoTracker green ratio increased in the TM group,. Representative images; n=4. * p ≤ 0.05, ** p ≤ 0.01, *** p ≤ 0.001 & **** p ≤ 0.0001 Student’s t-test.

**I)** Mitochondria volume fraction (V_V_), IMM/OMM ratio (S_A_) and Surface area (S_A_) of ER membrane and mitochondria calculated from TEM sections from a population of myoblasts and myotubes. * p ≤ 0.05, ** p ≤ 0.01, *** p ≤ 0.001 & **** p ≤ 0.0001 One-way ANOVA.

**J)** Heatmap from GSEA of mouse mitochondrial proteins significantly enriched in the upregulated proteins in the TM group compared to CNT.

**K)** GOBP enrichment dot plot from GSEA of mouse mitochondrial proteins significantly enriched in the upregulated proteins from the TM group compared to CNT.

**L)** Gene expression levels of *Kdelr2* were assessed using ΔΔCT method by qPCR, and normalised against *β2 microglobulin* (B2M) housekeeper gene. * p ≤ 0.05 One-way ANOVA.

**M)** Protein expression levels of ULK1 were assessed by Western Blot. Each lane represents independent biological replicates, data represented as mean SEM. * p ≤ 0.05 One-way ANOVA.

**N)** Protein expression levels of ATG5 were assessed by Western Blot, and normalised against total protein (Ponceau staining). Each lane represents independent biological replicates, data represented as mean SEM. * p ≤ 0.05 One-way ANOVA.

**O)** Amplitude values of [Ca^2+^]i increase show that the TM treated myoblasts exhibited a slightly augmented Store-Operated-Calcium-Entry (SOCE) compared to control. Student’s t-test.

**P)** The area fraction of the ER surface against the total area of the cells. Representative images; green: ER mNeon Green, blue: DAPI; n=3. Scale 275 μm. ** p ≤ 0.01 Student’s t-test.

**
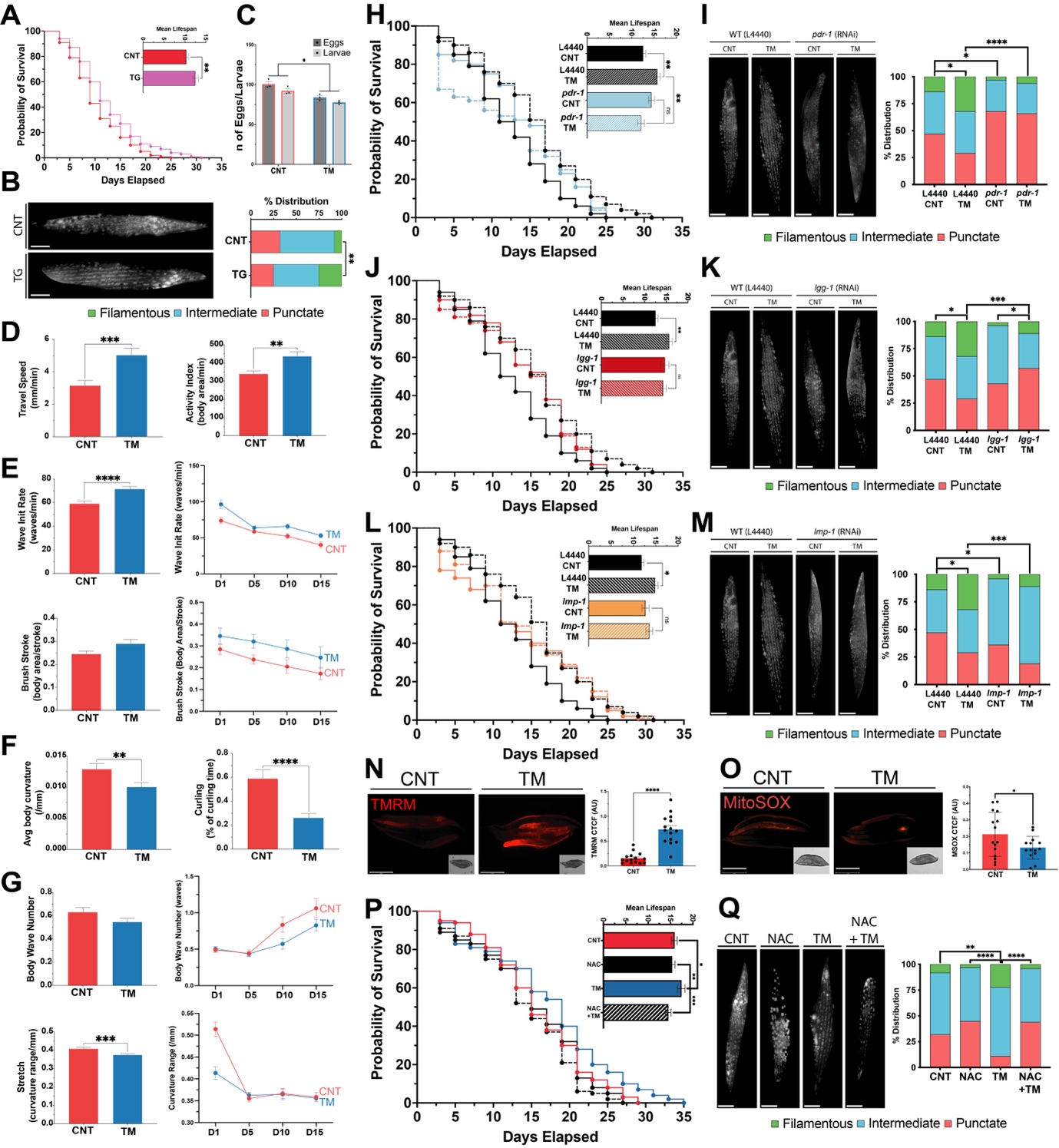
**

**Suppl. Figure 3:** **Early-life induction of UPR^ER^ in *C. elegans* activates autophagy and promotes physiological adaptations.**

**A)** Lifespan assay of TG treated N2 strain. Kaplan–Meier survival plots of two independent experiments initiated with 100 animals/group. Mean +/- SEM; **** p ≤ 0.0001 Log-rank (Mantel-Cox) test.

**B)** TG treated p*myo-3::mitogfp* reporter strain for muscle mitochondrial morphology, classified as either filamentous, intermediate or punctate. 3 independent experiments were initiated with 15 animals/ condition. Scale 50μm. n=3 * p<0.05 Chi-square.

**C)** Number of laid embryos compared to number of viable larvae in N2 worms treated with TM. * p ≤ 0.05 One-way ANOVA.

**D & E)** CeleST physical fitness parameters that decrease with age; average fitness parameters of days 1, 5, 10, & 15. Mean +/- SEM; **** p ≤ 0.0001 Student’s t-test.

**F & G)** CeleST physical fitness parameters that increase with age; average fitness parameters of days 1, 5, 10, & 15. Mean +/- SEM; *** p ≤ 0.001 Student’s t-test.

**H, J & L)** Lifespan assay of TM treated N2 strain and RNAi for *pdr-1, lgg-1* and *lmp-1* genes. Kaplan–Meier survival plots of two independent experiments initiated with 100 animals/group. Mean +/- SEM; **** p ≤ 0.0001 Log-rank (Mantel-Cox) test.

**I, K & M)** TM treated p*myo-3::mitogfp* reporter strain and RNAi for *pdr-1, lgg-1* and *lmp-1* genes. Representative images of reporter for muscle mitochondrial morphology, classified as either filamentous, intermediate or punctate. 3 independent experiments were initiated with 15 animals/ condition. Scale 50μm. n=3 * p<0.05 Chi-square.

**N)** N2 Wild-type worms incubated with TMRM dye, an indicator of mitochondrial membrane potential. Representative images of three independent experiments initiated with 15 animals/ strain; red: TMRM. Scale 275μm. Error bars SEM; * p ≤ 0.05 Student’s t-test.

**O)** N2 Wild-type worms incubated with MitoSOX dye, an indicator of mitochondrial ROS production. Representative images of three independent experiments initiated with 15 animals/ strain; red: MitoSOX. Scale 275μm. Error bars SEM; * p ≤ 0.05 Student’s t-test.

**P)** Lifespan assay of TM treated N2 strain and the antioxidant NAC. Kaplan–Meier survival plots of two independent experiments initiated with 100 animals/group. Mean +/- SEM; **** p ≤ 0.0001 Log-rank (Mantel-Cox) test.

**Q)** TM treated p*myo-3::mitogfp* reporter strain and the antioxidant NAC. Representative images of the reporter for muscle mitochondrial morphology, classified as either filamentous, intermediate or punctate. 3 independent experiments were initiated with 15 animals/ condition. Scale 50μm. n=3 * p<0.05 Chi-square.


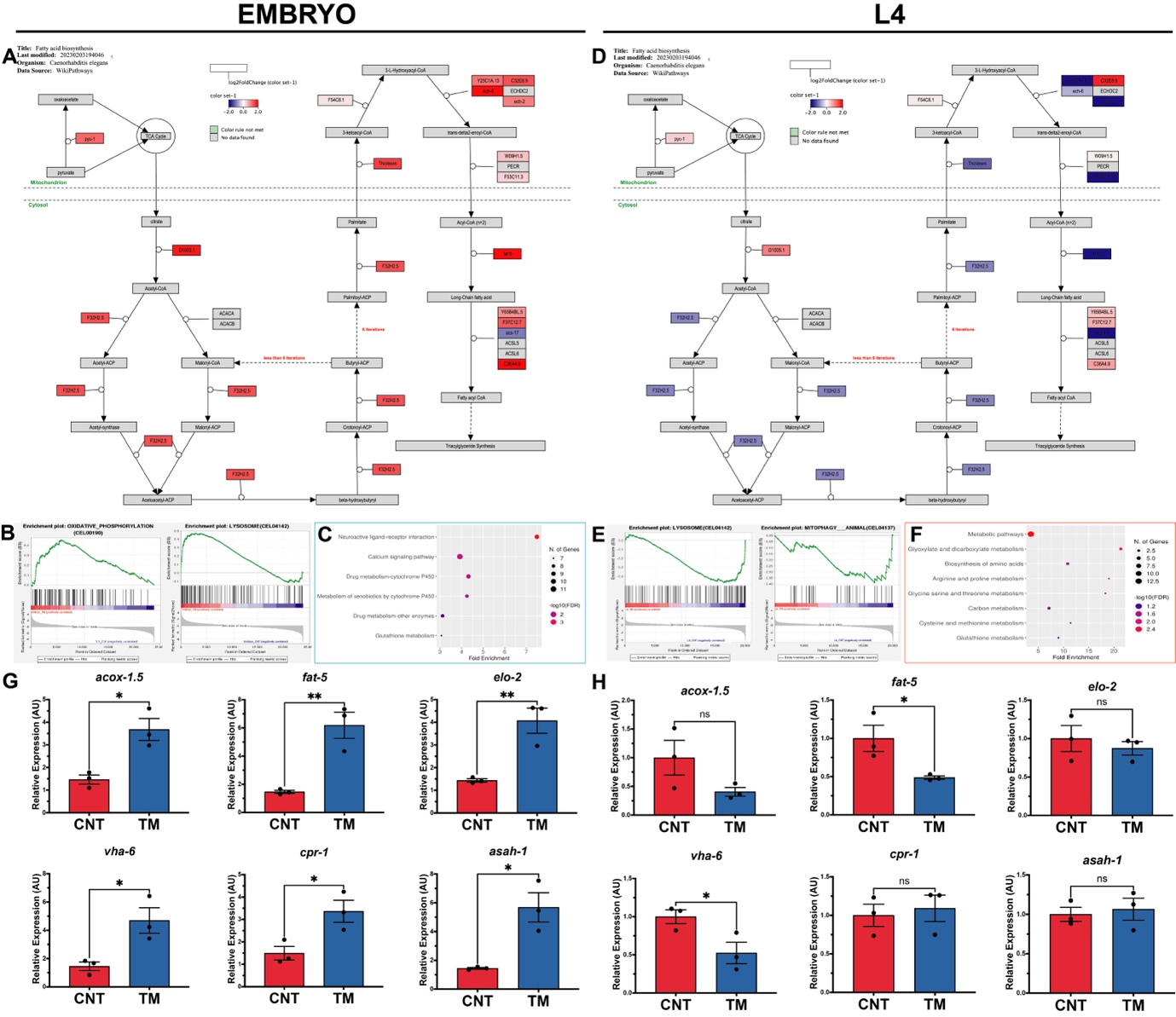


**Suppl. Figure 4:** **Early-life TM treatment of *C. elegans* embryos or at L4 stage promotes opposite effects on lipid metabolism and lysosomal function.**

**A)** PathVisio pathway analysis of EMBRYO DEGs revealed an upregulation of proteins involved in fatty acid biosynthesis in worms treated with TM.

**B)** GSEA pathways that are significantly enriched following treatment of embryos.

**C)** Dot plot of Shiny GO KEGG analysis of EMBRYO DEGs. Significantly downregulated pathways’ dotplot outlined in blue.

**D)** PathVisio pathway analysis of L4 DEGs revealed the downregulation of proteins involved in fatty acid biosynthesis in worms treated with TM.

**E)** GSEA pathways that are significantly enriched following treatment of L4 worms.

**F)** Dot plot of Shiny GO KEGG analysis of L4 DEGs. Significantly upregulated pathways’ dotplot outlined in red.

**G & H)** Gene expression levels of *acox-1.5, elo-2, fat-5, vha-6, asah-1 and cpr-1* were assessed using ΔΔCT method by qPCR, and normalised against *cdc-42* and *pmp-3* housekeeper genes. * p ≤ 0.05 One-way ANOVA.


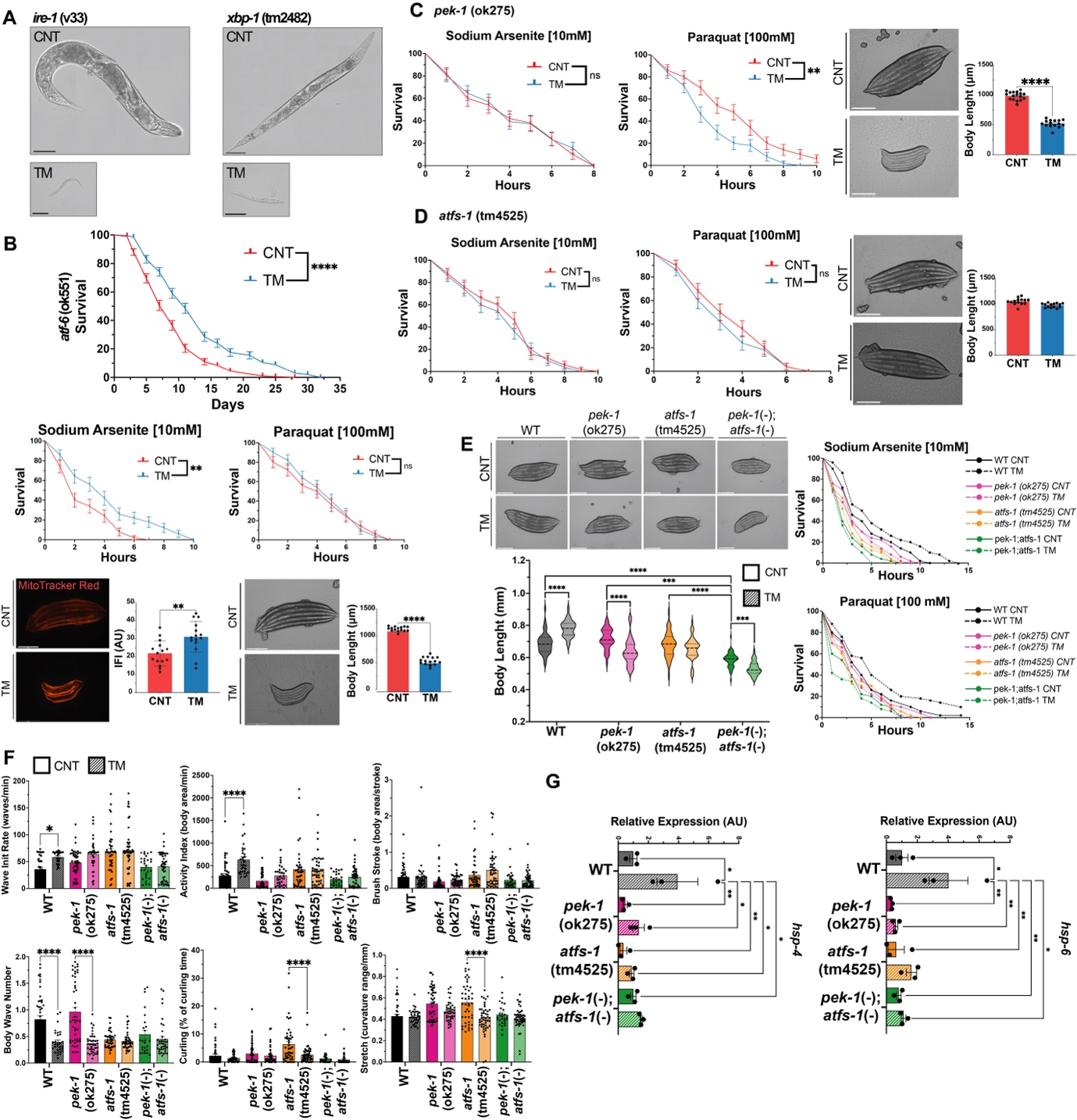


**Suppl.** **Figure 5:** E**arly-life TM treatment of *C. elegans* requires the PEK-1 arm of the UPR^ER^ for the beneficial adaptive response.**

**A)** Representative transmitted light images of TM treated *ire-1* (v33) worms and *xbp-1*(tm2482) worms.

**B)** TM treatment of *atf-6* (ok551) increased lifespan at 20°C. Kaplan–Meier survival plots of two independent experiments initiated with 100 animals per group, Log-rank (Mantel-Cox) test, **** p ≤ 0.0001. Increased survival of TM-treated *atf-6*(ok551) in 10 mM Sodium Arsenite and no effect on survival in 100 mM Paraquat compared to control. Kaplan–Meier survival plots of two independent experiments initiated with 50 animals per group by Log-rank (Mantel-Cox) test, ** p ≤ 0.01. Representative MitoTracker Red staining of adult day 1 *atf-6*(ok551) worms treated with TM. Scale 275 μm. Mean +/- SEM; ** p ≤ 0.01 Student’s t-test. Representative transmitted light images of adult day 1 *atf-6*(ok551) mutant worms. Scale 275μm. Error bars SEM; ** p ≤ 0.01 Student’s t-test.

**C)** TM treatment of *pek-1*(ok275) did not affect survival in 10 mM Sodium Arsenite and decreased survival in 100 mM Paraquat compared to control. Kaplan–Meier survival plots of two independent experiments were initiated with 50 animals per group using log-rank (Mantel-Cox) test, ** p ≤ 0.01. Representative transmitted light images of adult day 1 *pek-1*(ok275) worms. Scale 275 μm. Mean +/- SEM; **** p ≤ 0.0001 Student’s t-test.

**D)** TM treatment of *atfs-1*(tm4525) had no effect on survival in 10 mM Sodium Arsenite and 100 mM Paraquat compared to controls. Kaplan–Meier survival plots of two independent experiments initiated with 50 animals per group by Log-rank (Mantel-Cox) test. Representative transmitted light images of adult day 1 *atfs-1*(tm4525) worms. Scale 275μm. Mean +/- SEM; Student’s t-test.

**E)** Body length of adult day 1 worms following TM treatment in N2, *pek-1*(ok275), *atfs-1*(tm4525) and *pek-1;atfs-1* strains. Mean +/- SEM of 45 animals per assay. *** p ≤ 0.001 & **** p ≤ 0.0001 One-way ANOVA.

**F)** CeleST physical fitness parameters that change with age; average fitness parameters of day 1 TM-treated N2, *pek-1*, *atfs-1*, and *pek-1;atfs-1* mutant strains. Data from three independent experiments initiated with at least 10 animals per condition. Scale 275μm. Mean +/- SEM; * p ≤ 0.05 & **** p ≤ 0.0001 One-way ANOVA.

**G)** Gene expression levels of *hsp-4 and hsp-6* were assessed in N2, *pek-1*(ok275), *atfs-1*(tm4525) and *pek-1;atfs-1* strains using ΔΔCT method by qPCR, and normalised against *cdc-42* housekeeper gene. * p ≤ 0.05 One-way ANOVA.

**Supplementary Tables**

**Suppl.** **Table 1: Multiple comparison lifespan analysis at 20°C of wild-type (WT), *pek-1*(ok275) (PEK), *atfs-1(*tm4525) (ATFS) and *pek-1;atfs-1*.** Worms were treated with 1.25 μg/ml TM at EMBRYO stage during 24h, two independent experiments initiated with 100 animals per group.

| Condition | χ² | p-value | Corrected p-value |
| --- | --- | --- | --- |
| WT_C vs. WT_T | 12.43 | 0.0004 | 0.003 |
| WT_C vs. PEK_C | 8.14 | 0.0043 | 0.0304 |
| WT_C vs. PEK_T | 25.41 | 4.60E-07 | 0.0000032 |
| WT_C vs. ATFS_C | 3.75 | 0.0529 | 0.3706 |
| WT_C vs. ATFS_T | 12.44 | 0.0004 | 0.0029 |
| WT_C vs. PA_C | 22.09 | 0.0000026 | 0.000018 |
| WT_C vs. PA_T | 55.52 | 0 | 0 |
| WT_T vs. WT_C | 12.43 | 0.0004 | 0.003 |
| WT_T vs. PEK_C | 34.68 | 3.90E-09 | 2.70E-08 |
| WT_T vs. PEK_T | 62.85 | 0 | 0 |
| WT_T vs. ATFS_C | 26.06 | 3.30E-07 | 0.0000023 |
| WT_T vs. ATFS_T | 43.42 | 0 | 0 |
| WT_T vs. PA_C | 57.75 | 0 | 0 |
| WT_T vs. PA_T | 103.13 | 0 | 0 |
| PEK_C vs. WT_C | 8.14 | 0.0043 | 0.0304 |
| PEK_C vs. WT_T | 34.68 | 3.90E-09 | 2.70E-08 |
| PEK_C vs. PEK_T | 5.49 | 0.0191 | 0.1336 |
| PEK_C vs. ATFS_C | 0.73 | 0.3938 | 1 |
| PEK_C vs. ATFS_T | 0.53 | 0.4661 | 1 |
| PEK_C vs. PA_C | 3.83 | 0.0502 | 0.3516 |
| PEK_C vs. PA_T | 22.54 | 0.0000021 | 0.000014 |
| PEK_T vs. WT_C | 25.41 | 4.60E-07 | 0.0000032 |
| PEK_T vs. WT_T | 62.85 | 0 | 0 |
| PEK_T vs. PEK_C | 5.49 | 0.0191 | 0.1336 |
| PEK_T vs. ATFS_C | 12.4 | 0.0004 | 0.003 |
| PEK_T vs. ATFS_T | 4.15 | 0.0416 | 0.2911 |
| PEK_T vs. PA_C | 0.3 | 0.5815 | 1 |
| PEK_T vs. PA_T | 9.8 | 0.0017 | 0.0122 |
| ATFS_C vs. WT_C | 3.75 | 0.0529 | 0.3706 |
| ATFS_C vs. WT_T | 26.06 | 3.30E-07 | 0.0000023 |
| ATFS_C vs. PEK_C | 0.73 | 0.3938 | 1 |
| ATFS_C vs. PEK_T | 12.4 | 0.0004 | 0.003 |
| ATFS_C vs. ATFS_T | 3.56 | 0.0592 | 0.4143 |
| ATFS_C vs. PA_C | 9.51 | 0.002 | 0.0143 |
| ATFS_C vs. PA_T | 35.32 | 2.80E-09 | 2.00E-08 |
| ATFS_T vs. WT_C | 12.44 | 0.0004 | 0.0029 |
| ATFS_T vs. WT_T | 43.42 | 0 | 0 |
| ATFS_T vs. PEK_C | 0.53 | 0.4661 | 1 |
| ATFS_T vs. PEK_T | 4.15 | 0.0416 | 0.2911 |
| ATFS_T vs. ATFS_C | 3.56 | 0.0592 | 0.4143 |
| ATFS_T vs. PA_C | 1.96 | 0.1612 | 1 |
| ATFS_T vs. PA_T | 21.24 | 0.0000041 | 0.000028 |
| PA_C vs. WT_C | 22.09 | 0.0000026 | 0.000018 |
| PA_C vs. WT_T | 57.75 | 0 | 0 |
| PA_C vs. PEK_C | 3.83 | 0.0502 | 0.3516 |
| PA_C vs. PEK_T | 0.3 | 0.5815 | 1 |
| PA_C vs. ATFS_C | 9.51 | 0.002 | 0.0143 |
| PA_C vs. ATFS_T | 1.96 | 0.1612 | 1 |
| PA_C vs. PA_T | 11.29 | 0.0008 | 0.0055 |
| PA_T vs. WT_C | 55.52 | 0 | 0 |
| PA_T vs. WT_T | 103.13 | 0 | 0 |
| PA_T vs. PEK_C | 22.54 | 0.0000021 | 0.000014 |
| PA_T vs. PEK_T | 9.8 | 0.0017 | 0.0122 |
| PA_T vs. ATFS_C | 35.32 | 2.80E-09 | 2.00E-08 |
| PA_T vs. ATFS_T | 21.24 | 0.0000041 | 0.000028 |
| PA_T vs. PA_C | 11.29 | 0.0008 | 0.0055 |

**Suppl. Table 2: Multiple comparison Sodium Arsenite survival analysis at 20°C of wild-type (N2), *pek-1*(ok275) (PEK), *atfs-1*(tm4525) (ATFS) and pek-1;atfs-1.** Worms were treated with 1.25 μg/ml TM EMBRYO stage during 24h, two independent experiments initiated with 50 animals per group.

| Condition | χ² | p-value | Corrected p-value |
| --- | --- | --- | --- |
| WT_C vs. WT_T | 4.48 | 0.0343 | 0.2402 |
| WT_C vs. PEK_C | 1.59 | 0.2067 | 1 |
| WT_C vs. PEK_T | 5.09 | 2.41E-02 | 0.1685 |
| WT_C vs. ATFS_C | 6.57 | 0.0104 | 0.0727 |
| WT_C vs. ATFS_T | 10.73 | 0.0011 | 0.0074 |
| WT_C vs. PA_C | 14.43 | 0.0001 | 0.001 |
| WT_C vs. PA_T | 21.66 | 0.0000032 | 0.000023 |
| WT_T vs. WT_C | 4.48 | 0.0343 | 0.2402 |
| WT_T vs. PEK_C | 8.27 | 4.00E-03 | 2.82E-02 |
| WT_T vs. PEK_T | 13.18 | 0.0003 | 0.002 |
| WT_T vs. ATFS_C | 15.31 | 1.00E-04 | 0.0006 |
| WT_T vs. ATFS_T | 20.87 | 0.0000049 | 0.000034 |
| WT_T vs. PA_C | 26.91 | 2.10E-07 | 0.0000015 |
| WT_T vs. PA_T | 36.73 | 0 | 0 |
| PEK_C vs. WT_C | 1.59 | 0.2067 | 1 |
| PEK_C vs. WT_T | 8.27 | 4.00E-03 | 2.82E-02 |
| PEK_C vs. PEK_T | 1.46 | 0.2267 | 1 |
| PEK_C vs. ATFS_C | 2.77 | 0.0961 | 0.6726 |
| PEK_C vs. ATFS_T | 6.2 | 0.0128 | 0.0897 |
| PEK_C vs. PA_C | 8.27 | 0.004 | 0.0283 |
| PEK_C vs. PA_T | 13.96 | 0.0002 | 0.0013 |
| PEK_T vs. WT_C | 5.09 | 2.41E-02 | 0.1685 |
| PEK_T vs. WT_T | 13.18 | 0.0003 | 0.002 |
| PEK_T vs. PEK_C | 1.46 | 0.2267 | 1 |
| PEK_T vs. ATFS_C | 0.27 | 0.6021 | 1 |
| PEK_T vs. ATFS_T | 1.86 | 0.1721 | 1 |
| PEK_T vs. PA_C | 3.23 | 0.0722 | 0.5057 |
| PEK_T vs. PA_T | 8.5 | 0.0035 | 0.0248 |
| ATFS_C vs. WT_C | 6.57 | 0.0104 | 0.0727 |
| ATFS_C vs. WT_T | 15.31 | 1.00E-04 | 0.0006 |
| ATFS_C vs. PEK_C | 2.77 | 0.0961 | 0.6726 |
| ATFS_C vs. PEK_T | 0.27 | 0.6021 | 1 |
| ATFS_C vs. ATFS_T | 1 | 0.3165 | 1 |
| ATFS_C vs. PA_C | 2.3 | 0.1296 | 0.9072 |
| ATFS_C vs. PA_T | 7.55 | 6.00E-03 | 4.20E-02 |
| ATFS_T vs. WT_C | 10.73 | 0.0011 | 0.0074 |
| ATFS_T vs. WT_T | 20.87 | 0.0000049 | 0.000034 |
| ATFS_T vs. PEK_C | 6.2 | 0.0128 | 0.0897 |
| ATFS_T vs. PEK_T | 1.86 | 0.1721 | 1 |
| ATFS_T vs. ATFS_C | 1 | 0.3165 | 1 |
| ATFS_T vs. PA_C | 0.32 | 0.5729 | 1 |
| ATFS_T vs. PA_T | 3.26 | 0.0708 | 0.4957 |
| PA_C vs. WT_C | 14.43 | 0.0001 | 0.001 |
| PA_C vs. WT_T | 26.91 | 2.10E-07 | 0.0000015 |
| PA_C vs. PEK_C | 8.27 | 0.004 | 0.0283 |
| PA_C vs. PEK_T | 3.23 | 0.0722 | 0.5057 |
| PA_C vs. ATFS_C | 2.3 | 0.1296 | 0.9072 |
| PA_C vs. ATFS_T | 0.32 | 0.5729 | 1 |
| PA_C vs. PA_T | 1.63 | 0.2014 | 1 |
| PA_T vs. WT_C | 21.66 | 0.0000032 | 0.000023 |
| PA_T vs. WT_T | 36.73 | 0 | 0 |
| PA_T vs. PEK_C | 13.96 | 0.0002 | 0.0013 |
| PA_T vs. PEK_T | 8.5 | 0.0035 | 0.0248 |
| PA_T vs. ATFS_C | 7.55 | 6.00E-03 | 4.20E-02 |
| PA_T vs. ATFS_T | 3.26 | 0.0708 | 0.4957 |
| PA_T vs. PA_C | 1.63 | 0.2014 | 1 |

**Suppl. Table 3: Multiple comparison Paraquat survival analysis at 20°C of N2 wild-type (WT), *pek-1*(ok275) (PEK), *atfs-1(*tm4525) (ATFS) and *pek-1*;*atfs-1* strains.** Worms were treated with 1.25 μg/ml TM EMBRYO stage during 24h, two independent experiments initiated with 50 animals per group.

| Condition | χ² | p-value | Corrected p-value |
| --- | --- | --- | --- |
| WT_C vs. WT_T | 4.13 | 0.0422 | 0.2956 |
| WT_C vs. PEK_C | 0.55 | 0.4593 | 1 |
| WT_C vs. PEK_T | 3.58 | 5.84E-02 | 0.4088 |
| WT_C vs. ATFS_C | 1.97 | 0.1606 | 1 |
| WT_C vs. ATFS_T | 2.98 | 0.0846 | 0.5919 |
| WT_C vs. PA_C | 5.34 | 0.0208 | 0.1456 |
| WT_C vs. PA_T | 10.29 | 0.0013 | 0.0094 |
| WT_T vs. WT_C | 4.13 | 0.0422 | 0.2956 |
| WT_T vs. PEK_C | 7.14 | 7.50E-03 | 5.27E-02 |
| WT_T vs. PEK_T | 11.47 | 0.0007 | 0.0049 |
| WT_T vs. ATFS_C | 9.42 | 2.10E-03 | 0.015 |
| WT_T vs. ATFS_T | 10.53 | 0.0012 | 0.0082 |
| WT_T vs. PA_C | 13.53 | 0.0002 | 0.0016 |
| WT_T vs. PA_T | 21.12 | 0.0000043 | 0.00003 |
| PEK_C vs. WT_C | 0.55 | 0.4593 | 1 |
| PEK_C vs. WT_T | 7.14 | 7.50E-03 | 5.27E-02 |
| PEK_C vs. PEK_T | 1.72 | 0.1903 | 1 |
| PEK_C vs. ATFS_C | 0.77 | 0.3815 | 1 |
| PEK_C vs. ATFS_T | 1.47 | 0.2246 | 1 |
| PEK_C vs. PA_C | 2.71 | 0.0999 | 0.699 |
| PEK_C vs. PA_T | 6.92 | 0.0085 | 0.0595 |
| PEK_T vs. WT_C | 3.58 | 5.84E-02 | 0.4088 |
| PEK_T vs. WT_T | 11.47 | 0.0007 | 0.0049 |
| PEK_T vs. PEK_C | 1.72 | 0.1903 | 1 |
| PEK_T vs. ATFS_C | 0.37 | 0.5422 | 1 |
| PEK_T vs. ATFS_T | 0.08 | 0.7726 | 1 |
| PEK_T vs. PA_C | 0.22 | 0.6361 | 1 |
| PEK_T vs. PA_T | 2.56 | 0.1095 | 0.7668 |
| ATFS_C vs. WT_C | 1.97 | 0.1606 | 1 |
| ATFS_C vs. WT_T | 9.42 | 2.10E-03 | 0.015 |
| ATFS_C vs. PEK_C | 0.77 | 0.3815 | 1 |
| ATFS_C vs. PEK_T | 0.37 | 0.5422 | 1 |
| ATFS_C vs. ATFS_T | 0.1 | 0.754 | 1 |
| ATFS_C vs. PA_C | 1.12 | 0.2905 | 1 |
| ATFS_C vs. PA_T | 4.65 | 3.10E-02 | 2.17E-01 |
| ATFS_T vs. WT_C | 2.98 | 0.0846 | 0.5919 |
| ATFS_T vs. WT_T | 10.53 | 0.0012 | 0.0082 |
| ATFS_T vs. PEK_C | 1.47 | 0.2246 | 1 |
| ATFS_T vs. PEK_T | 0.08 | 0.7726 | 1 |
| ATFS_T vs. ATFS_C | 0.1 | 0.754 | 1 |
| ATFS_T vs. PA_C | 0.7 | 0.4024 | 1 |
| ATFS_T vs. PA_T | 4.1 | 0.043 | 0.3008 |
| PA_C vs. WT_C | 5.34 | 0.0208 | 0.1456 |
| PA_C vs. WT_T | 13.53 | 0.0002 | 0.0016 |
| PA_C vs. PEK_C | 2.71 | 0.0999 | 0.699 |
| PA_C vs. PEK_T | 0.22 | 0.6361 | 1 |
| PA_C vs. ATFS_C | 1.12 | 0.2905 | 1 |
| PA_C vs. ATFS_T | 0.7 | 0.4024 | 1 |
| PA_C vs. PA_T | 1.16 | 0.282 | 1 |
| PA_T vs. WT_C | 10.29 | 0.0013 | 0.0094 |
| PA_T vs. WT_T | 21.12 | 0.0000043 | 0.00003 |
| PA_T vs. PEK_C | 6.92 | 0.0085 | 0.0595 |
| PA_T vs. PEK_T | 2.56 | 0.1095 | 0.7668 |
| PA_T vs. ATFS_C | 4.65 | 3.10E-02 | 2.17E-01 |
| PA_T vs. ATFS_T | 4.1 | 0.043 | 0.3008 |
| PA_T vs. PA_C | 1.16 | 0.282 | 1 |

**Suppl. Table 4: Multiple comparison lifespan analysis at 20°C of wild-type L4440 CNT, L4440 TM, RNAi *lgg-1* CNT and RNAi *lgg-1* TM.** Worms were treated with 1.25 μg/ml TM at EMBRYO stage during 24h, experiments were initiated with 100 animals per group.

| Condition | χ² | p-value | Corrected p-value |
| --- | --- | --- | --- |
| L4440_CNT vs. L4440_TM | 15.25 | 1.00E-04 | 0.0003 |
| L4440_CNT vs. lgg-1_CNT | 5.23 | 0.0222 | 0.0665 |
| L4440_CNT vs. lgg-1_TM | 7.02 | 8.10E-03 | 2.42E-02 |
| L4440_TM vs. L4440_CNT | 15.25 | 0.0001 | 0.0003 |
| L4440_TM vs. lgg-1_CNT | 1.34 | 0.2471 | 0.7414 |
| L4440_TM vs. lgg-1_TM | 2.45 | 1.18E-01 | 0.3534 |
| lgg-1_CNT vs. L4440_CNT | 5.23 | 0.0222 | 0.0665 |
| lgg-1_CNT vs. L4440_TM | 1.34 | 2.47E-01 | 0.7414 |
| lgg-1_CNT vs. lgg-1_TM | 0.01 | 9.35E-01 | 1.00E+00 |
| lgg-1_TM vs. L4440_CNT | 7.02 | 8.10E-03 | 2.42E-02 |
| lgg-1_TM vs. L4440_TM | 2.45 | 1.18E-01 | 3.53E-01 |
| lgg-1_TM vs. lgg-1_CNT | 0.01 | 0.9354 | 1 |

**Suppl. Table 5: Multiple comparison lifespan analysis at 20°C of wild-type L4440 CNT, L4440 TM, RNAi *pdr-1* CNT and RNAi *pdr-1* TM.** Worms were treated with 1.25 μg/ml TM at EMBRYO stage during 24h, experiments were initiated with 100 animals per group.

| Condition | χ² | p-value | Corrected p-value |
| --- | --- | --- | --- |
| L4440_CNT vs. L4440_TM | 15.25 | 1.00E-04 | 0.0003 |
| L4440_CNT vs. pdr-1_CNT | 7.62 | 0.0058 | 0.0173 |
| L4440_CNT vs. pdr-1_TM | 1.33 | 2.49E-01 | 7.48E-01 |
| L4440_TM vs. L4440_CNT | 15.25 | 0.0001 | 0.0003 |
| L4440_TM vs. pdr-1_CNT | 2.11 | 0.1459 | 0.4377 |
| L4440_TM vs. pdr-1_TM | 6.33 | 1.19E-02 | 0.0357 |
| pdr-1_CNT vs. L4440_CNT | 7.62 | 0.0058 | 0.0173 |
| pdr-1_CNT vs. L4440_TM | 2.11 | 1.46E-01 | 0.4377 |
| pdr-1_CNT vs. pdr-1_TM | 1.11 | 2.91E-01 | 8.74E-01 |
| pdr-1_TM vs. L4440_CNT | 1.33 | 2.49E-01 | 7.48E-01 |
| pdr-1_TM vs. L4440_TM | 6.33 | 1.19E-02 | 3.57E-02 |
| pdr-1_TM vs. pdr-1_CNT | 1.11 | 0.2912 | 0.8737 |

**Suppl. Table 6: Multiple comparison lifespan analysis at 20°C of wild-type L4440 CNT, L4440 TM, RNAi *lmp-1* CNT and RNAi *lmp-1* TM.** Worms were treated with 1.25 μg/ml TM at EMBRYO stage during 24h, experiments were initiated with 100 animals per group.

| Condition | χ² | p-value | Corrected p-value |
| --- | --- | --- | --- |
| L4440_CNT vs. L4440_TM | 15.25 | 1.00E-04 | 0.0003 |
| L4440_CNT vs. lmp-1_CNT | 5.45 | 0.0196 | 0.0587 |
| L4440_CNT vs. lmp-1_TM | 8.68 | 3.20E-03 | 9.60E-03 |
| L4440_TM vs. L4440_CNT | 15.25 | 0.0001 | 0.0003 |
| L4440_TM vs. lmp-1_CNT | 1.1 | 0.2944 | 0.8831 |
| L4440_TM vs. lmp-1_TM | 0.39 | 5.33E-01 | 1 |
| lmp-1_CNT vs. L4440_CNT | 5.45 | 0.0196 | 0.0587 |
| lmp-1_CNT vs. L4440_TM | 1.1 | 2.94E-01 | 0.8831 |
| lmp-1_CNT vs. lmp-1_TM | 0.35 | 5.55E-01 | 1.00E+00 |
| lmp-1_TM vs. L4440_CNT | 8.68 | 3.20E-03 | 9.60E-03 |
| lmp-1_TM vs. L4440_TM | 0.39 | 5.33E-01 | 1.00E+00 |
| lmp-1_TM vs. lmp-1_CNT | 0.35 | 0.5554 | 1 |

**Suppl. Table 7: Multiple comparison lifespan analysis at 20°C of wild-type CNT, NAC, TM and NAC+TM.** Worms were treated with 1.25 μg/ml TM at EMBRYO stage during 24h, experiments were initiated with 100 animals per group.

| Condition | χ² | p-value | Corrected p-value |
| --- | --- | --- | --- |
| CNT vs. NAC | 0.08 | 7.81E-01 | 1.000 |
| CNT vs. TM | 5.64 | 0.0176 | 0.053 |
| CNT vs. NAC+TM | 3.2 | 7.34E-02 | 0.220 |
| NAC vs. CNT | 0.08 | 0.7814 | 1.000 |
| NAC vs. TM | 7.07 | 0.0078 | 0.024 |
| NAC vs. NAC+TM | 2.45 | 1.18E-01 | 0.353 |
| TM vs. CNT | 5.64 | 0.0176 | 0.053 |
| TM vs. NAC | 7.07 | 7.80E-03 | 0.024 |
| TM vs. NAC+TM | 14.83 | 1.00E-04 | 0.000 |
| NAC+TM vs. CNT | 3.2 | 7.34E-02 | 0.220 |
| NAC+TM vs. NAC | 2.45 | 1.18E-01 | 0.353 |
| NAC+TM vs. TM | 14.83 | 0.0001 | 0.000 |

**Suppl. Table 8: Key resources table.**

| REAGENT or RESOURCE | SOURCE | IDENTIFIER |
| --- | --- | --- |
| Antibodies | | |
| mouse anti-CHOP | Cell Signaling Technology | 2895 |
| mouse anti-MF20 | Developmental Studies Hybridoma Bank | MF 20 |
| rabbit anti-ATF4 | Cell Signaling Technology | 11815 |
| rabbit anti-eIF2α | Cell Signaling Technology | 5324 |
| rabbit anti-phospo-eIF2α | Cell Signaling Technology | 3398 |
| rabbit anti-MFN2 | Cell Signaling Technology | 9482 |
| rabbit anti-TOM20 | Cell Signaling Technology | 42406 |
| rabbit anti-GRP78 | Cell Signaling Technology | 3177 |
| rabbit anti-ATG5 | Cell Signaling Technology | 12994 |
| rabbit anti-P62 | Cell Signaling Technology | 5114 |
| rabbit anti-LC3B | Cell Signaling Technology | 2775 |
| rabbit anti-ULK1 | Cell Signaling Technology | 8054 |
| Goat anti-Mouse IgG (H+L) Alexa FluorTM Plus 555 | Thermo Fisher Scientific | A32727 |
| Goat anti-Rabbit IgG (H+L) Alexa FluorTM 546 | Thermo Fisher Scientific | A-11035 |
| Bacterial and virus strains | | |
| *E. coli*: Strain OP50 | Caenorhabditis Genetics Center | OP50 |
| TOP10 Competent Cells | Thermo Fisher | TOP10 |
| Chemicals, peptides, and recombinant proteins | | |
| 2-Propanol | Merck | I9516 |
| 25X dNTP Mix (100 mM) | Thermo Fisher | 4368814 |
| 3 PrimeG Gradient Thermal Cycler | Techne | 93945-09 |
| 4′,6-diamidino-2-phenylindole (DAPI) | Merck | 268298 |
| Acetic acid | Sigma | A6283 |
| Acrylamide | Sigma | A3699 |
| Agar | Sigma | A1296 |
| Agarose Ultrapure | Thermo Fisher | 16500 |
| Ammonium Persulfate | Sigma | A3678 |
| Beta-mercaptoethanol | Sigma | M7522 |
| Bleach | Household |  |
| Bovine Serum Albumin BSA | Merck | A2153 |
| Bradford Reagent | Bio-Rad | 5000006 |
| Bromophenol blue | Sigma | 114391 |
| BSA | Sigma | A3059 |
| CaCl2 | Sigma | C1016 |
| Cell culture flask, T-175 | Sarstedt | 833912 |
| Cell culture flask, T-25 | Sarstedt | 833910 |
| Cell culture flask, T-75 | Sarstedt | 833911 |
| Cell culture plate, 12 well | Sarstedt | 833921 |
| Cell culture plate, 24 well | Sarstedt | 833922 |
| Cell culture plate, 6 well | Sarstedt | 833920 |
| Cell culture plate, 96 well | Sarstedt | 833924 |
| Cell scraper, sterile | Sarstedt | 833950 |
| Chloroform | Sigma | C0549 |
| Cholesterol | Sigma | C8667 |
| DMEM | Sigma | D5796 |
| EDTA | Sigma | ED2SS |
| EGTA | Sigma | E3889 |
| Electric Pestle | VWR | SCERSP749540-0000 |
| Ethanol | Sigma | E7023 |
| Fast SYBR™ Green Master Mix | Applied | 4385617 |
| FBS | Sigma | F7524 |
| Fisher BioReagents™ EZ-Run™ Pre-stained Rec Protein Ladder | Fisher Bioreagents | BP36031 |
| Glucose | Sigma | G7021 |
| Glutaraldehyde | Sigma | G5882 |
| Glycerol | Sigma | G6279 |
| Glycine | Sigma | G8898 |
| HEPES | Sigma | H3375 |
| H_2_O_2_ | Sigma | H1009 |
| Horse Serum | Thermo Fisher Scientific | 26050088 |
| Hydromount | Scientific laboratory supplies | D2176 |
| Isopropanol | Sigma | I9516 |
| KCl | Sigma | P3911 |
| K2HPO4 | Sigma | 3786 |
| KH2PO4 | Sigma | P9791 |
| Laminin | Merck | L2020 |
| Lead Citrate | Leica Biosystems | S534/2 |
| Lipofectamine 2000 Reagent | Thermo Fisher Scientific | 11668019 |
| Lipofectamine 3000 Reagent | Thermo Fisher Scientific | L3000001 |
| Methanol | Sigma | 34860 |
| MgCl2 | Sigma | M8266 |
| MgSO4 | Sigma | M7506 |
| N,N,N′,N′-Tetramethylethylenediamine | Merck | T9281 |
| Na2HPO4 | Sigma | S0876 |
| NaAsO2 | Sigma | S7400 |
| NaCl | Sigma | S9888 |
| NaOH | Sigma | S5881 |
| NEM | Sigma | E3876 |
| Nitrocellulose Blotting membrane | GE Healthcare Life Sciences | 1060003 |
| Nystatin | Sigma | N3503 |
| OsO4 | Sigma | 201030 |
| Paraformaldehyde | Thermo Fisher | 10131580 |
| Paraquat | Sigma | 856177 |
| PBS | Sigma | D8537 |
| PCR plate half skirt, 96 well, transparent, Low-Profile | Sarstedt | 721981232 |
| PCR single tube, 0.5 ml, Biosphere®plus | Sarstedt | 72735100 |
| Penicillin/Streptomycin | Thermo Fisher Scientific | 15140122 |
| Peptone | Sigma | 91249 |
| Phosphatase Inhibitor | Merck | P0044 |
| Ponceau S | Sigma | 141194 |
| Protease Inhibitor Cocktail | Sigma | P8340 |
| Protein Assay Dye Reagent Concentrate | Bio Rad | 5000006 |
| Random Hexamers (50 µM) | Thermo Fisher | N8080127 |
| REDTaq® ReadyMix™ PCR Reaction Mix | Merck | R2523 |
| RiboLock RNase Inhibitor (40U/µL) | Thermo Fisher | EO0381 |
| RNase free water | Sigma | W4502 |
| SDS | Sigma | L3771 |
| Sodium cacodylate trihydrate | Sigma | C0250 |
| Sodium Dodecyl Sulfate | Merck | L3771 |
| SuperScript™ II Reverse Transcriptase kit (includes 5X First- Strand Buffer and 0.1 M DTT) | Thermo Fisher | 18064014 |
| SYBR Green | Qiagen | 339347 |
| SYBR™ Safe DNA Gel Stain | Thermo Fisher | S33102 |
| tBuOOH | Sigma | 654833 |
| TEMED | Sigma | T9281 |
| TritonTM X-100 | Sigma | T8787 |
| Trizma® base | Sigma | T1503 |
| Trizma® hydrochloride | Merck | T3253 |
| TRIzol Reagent | Life Technologies | 15596018 |
| TrypLE™ Express Enzyme | Thermo Fisher Scientific | 12604013 |
| Tunicamycin | Sigma | T7765 |
| Thapsigargin | Sigma | 586005 |
| Tween 20 | Merck | P9416 |
| Uranyl Acetate | Leica Biosystems | S534/1 |
| Urea | Sigma | U5378 |
| Critical commercial assays | | |
| Fluo-4, AM | Thermo Fisher Scientific | F14201 |
| LysoSensor Green DND189 | Invitrogen | L7535 |
| LysoTracker Red DND99 | Invitrogen | L7528 |
| MitoTrackerTM Green FM | Thermo Fisher Scientific | M7514 |
| MitoTrackerTM Red CMXRos | Thermo Fisher Scientific | M7512 |
| MitoSOXTM Red | Thermo Fisher Scientific | M36008 |
| Oil Red O | Sigma | O0625 |
| PureYield™ Plasmid Miniprep System | Promega | A1223 |
| TMRM | Thermo Fisher Scientific | T668 |
| Deposited data | | |
| Original data | Original data | Original data |
| Proteomics data | Proteomics data | Proteomics data |
| RNA-seq data | RNA-seq data | RNA-seq data |
| Experimental models: Cell lines | | |
| C2C12 | ATCC | Cat # CRL-1772 |
| Experimental models: Organisms/strains | | |
| *C. elegans*: wild type | Caenorhabditis Genetics Center | N2 |
| *C. elegans*: zcIs4 [*hsp-4::GFP*] V | Dr Antonio Miranda Vizuete | SJ4005 |
| *C. elegans*: zcIs13 [*hsp-6p::GFP + lin-15 (+)*] V | Dr Antonio Miranda Vizuete | SJ4100 |
| *C. elegans*: zcIs14 [*myo-3::GFP (mit)*] | Caenorhabditis Genetics Center | SJ4103 |
| *C. elegans*: zu476 [*cox-4::eGFP::3xFLAG*] I | Caenorhabditis Genetics Center | JJ2586 |
| *C. elegans*: *unc-119 (ed3);Ex*[*_pmyo-3_TOMM-20::Rosella;unc-119 (+)*] | Professor Nektarios Tavernarakis Crete, Greece | IR2539 |
| *C. elegans*: *pek-1* (ok275) X | Caenorhabditis Genetics Center | RB545 |
| *C. elegans*: *atf-6* (ok551) X | Caenorhabditis Genetics Center | RB772 |
| *C. elegans: ire-1* (v33) II | Caenorhabditis Genetics Center | RE666 |
| *C. elegans*: *xbp-1* (tm2482) III | NBRP Shigen | TM2482 |
| *C. elegans*: *atfs-1* (tm4525) V | Dr Antonio Miranda Vizuete | VZ591 |
| *C. elegans*: *pek-1* (ok275) X; *atfs-1* (tm4525) V | Invertebrate Physiology Lab Galway | MCD10 |
| *C. elegans*: *pek-1 (ok275) X; zcIs14 [myo-3::GFP (mit)]* | Invertebrate Physiology Lab Galway | MCD18 |
| *C. elegans*: *atfs-1 (tm4525) V; zcIs14 [myo-3::GFP (mit)]* | Invertebrate Physiology Lab Galway | MCD19 |
| *C. elegans*: *pek-1 (ok275) X; atfs-1 (tm4525) V; zcIs14 [myo-3::GFP (mit)]* | Invertebrate Physiology Lab Galway | MCD20 |
| Oligonucleotides | | |
| F (Ms xbp1) tgtctattcggagtgggcct | Sigma | N/A |
| R (Ms xbp1) gaaccttgttgggaacgggag | Sigma | N/A |
| F (Ce xbp1u) ccgatccacctccatcaac | Sigma | N/A |
| R (Ce xbp1u) accgtctgctccttcctcaatg | Sigma | N/A |
| F (Ce xbp1s) tgcctttgaatcagcagtgg | Sigma | N/A |
| R (Ce xbp1s) accgtctgctccttcctcaatg | Sigma | N/A |
| OF (ok275) ctgagccatcgacaaactca | Sigma | N/A |
| IF (ok275) gcagaagagccacttaatgac | Sigma | N/A |
| OR (ok275) ccttggtaccattcaacgct | Sigma | N/A |
| OF (tm4525) cactgccaatgtgagtcatg | Sigma | N/A |
| IF (tm4525) ccgatcaatgaccagctgacc | Sigma | N/A |
| OR (tm4525) gtcggcaatgcgaaaaagat | Sigma | N/A |
| OF (v33) gtctcatcggcgaatagcct | Sigma | N/A |
| IF (v33) cgtctctgaatccaaagctctc | Sigma | N/A |
| OR (v33) ggctggagtacggttgacaaatg | Sigma | N/A |
| OF (tm2482) gccccatattcgctacactg | Sigma | N/A |
| IF (tm2482) gcaaagatcgaggatgtgatgc | Sigma | N/A |
| OR (tm2482) catgtggttgcatagaatcg | Sigma | N/A |
| OF (ok551) aatgaccaggaaatgtggga | Sigma | N/A |
| IF (ok551) gcaaacgaagaagtcggacc | Sigma | N/A |
| OR (ok551) aagtgtcaattggccagtcc | Sigma | N/A |
| OF (gk169) cgctcctcctaaacgttgtagtc | Sigma | N/A |
| IR (gk169) ggatattgaaacccaacggcaacg | Sigma | N/A |
| OR (gk169) ctagggatacgggggaaattag | Sigma | N/A |
| Recombinant DNA | | |
| mouse PERK siRNA | Ambion | 100418 |
| mouse CHOP siRNA | Ambion | 288791 |
| mouse PRDX1 siRNA | Life Technologies | 4390824 |
| mouse PRDX2 siRNA | Life Technologies | 4390824 |
| *pCLBW cox8 EGFP mCherry* | Addgene | 78520 |
| *ER (KDEL)-mNeonGreen* | Addgene | 137804 |
| Software and algorithms | | |
| Prism 9 | GraphPad Software | RRID:SCR_002798 |
| ImageJ | NIH | RRID:SCR_003070 |
| Seahorse Wave | Agilent Technologies | RRID:SCR_014526 |
| Image Studio Lite | Image Studio Lite | RRID:SCR_013715 |
| CeleST | Christophe Restif et al., 2014 | N/A |
| Other | | |
| EVOS M7000 Imaging System | Thermo Fisher | AMF7000 |
| Nanodrop 2000 | Thermo Fisher | N/A |
| Odyssey® Fc Imaging System | LI-COR Biosciences | OFC-1025 |
| omniPAGE Mini Vertical Protein Electrophoresis System | Cleaver Scientific | VS10 |
| Owl™ EasyCast™ B2 Mini Gel Electrophoresis Systems | Thermo Fisher | B2 |
| PowerEase™ 90W Power Supply (115 VAC) | Fisher Scientific | PS0090 |
| Seesaw-Rocker Large 230V | Stuart | 51900-32 |
| Sense Beta Plus Microplate Reader | Hidex | 425-311 |
| StepOnePlus™ Real-Time PCR System | Applied Biosystems | 4376600 |
| Transfer Pipette | Sarstedt | 861171 |

**Uncropped Western blot membranes:**

**
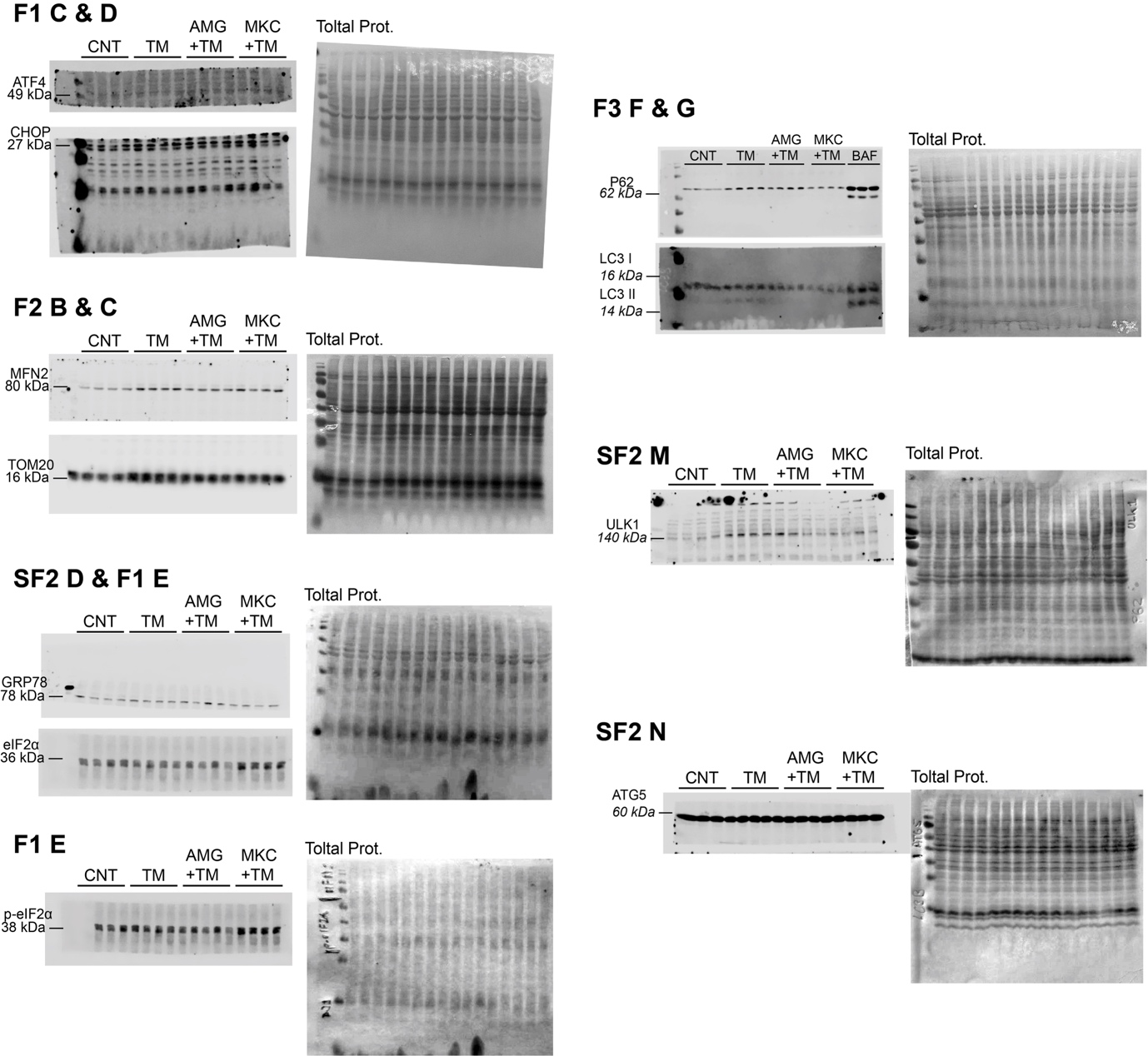
**

**Uncropped Western blots**. Uncropped membranes of the Western blot analysis shown in Figure 1, 2, 3 & Suppl. Figure 2.
